# Protease-Mediated *T*_1_ Contrast Enhancement of Multilayered Magneto-Gadolinium Nanostructures for Imaging and Magnetic Hyperthermia

**DOI:** 10.1021/acsami.3c13914

**Published:** 2024-01-31

**Authors:** Sahitya Kumar Avugadda, Nisarg Soni, Emille M. Rodrigues, Stefano Persano, Teresa Pellegrino

**Affiliations:** Nanomaterials for Biomedical Applications, Istituto Italiano di Tecnologia, 16163 Genova, Italy

**Keywords:** iron oxide nanocubes, gadolinium fluoride nanoparticles, magnetic hyperthermia, multifunctional, MRI dual imaging, enzyme responsive, *T*_1_ contrast agents

## Abstract

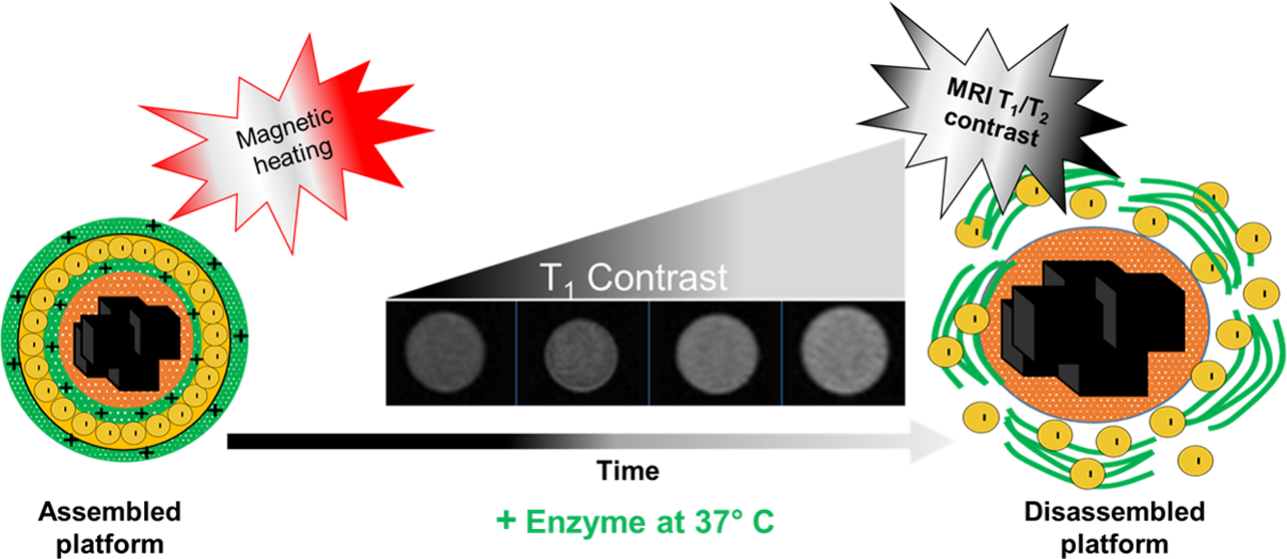

In this work, we constructed a multifunctional composite nanostructure for combined magnetic hyperthermia therapy and magnetic resonance imaging based on *T*_1_ and *T*_2_ signals. First, iron oxide nanocubes with a benchmark heating efficiency for magnetic hyperthermia were assembled within an amphiphilic polymer to form magnetic nanobeads. Next, poly(acrylic acid)-coated inorganic sodium gadolinium fluoride nanoparticles were electrostatically loaded onto the magnetic nanobead surface via a layer-by-layer approach by employing a positively charged enzymatic-cleavable biopolymer. The positive–negative multilayering process was validated through the changes occurring in surface ζ-potential values and structural characterization by transmission electron microscopy (TEM) imaging. These nanostructures exhibit an efficient heating profile, in terms of the specific absorption rates under clinically accepted magnetic field conditions. The addition of protease enzyme mediates the degradation of the surface layers of the nanostructures with the detachment of gadolinium nanoparticles from the magnetic beads and exposure to the aqueous environment. Such a process is associated with changes in the *T*_1_ relaxation time and contrast and a parallel decrease in the *T*_2_ signal. These structures are also nontoxic when tested on glioblastoma tumor cells up to a maximum gadolinium dose of 125 μg mL^–1^, which also corresponds to a iron dose of 52 μg mL^–1^. Nontoxic nanostructures with such enzyme-triggered release mechanisms and *T*_1_ signal enhancement are desirable for tracking tumor microenvironment release with remote *T*_1_-guidance and magnetic hyperthermia therapy actuation to be done at the diseased site upon verification of magnetic resonance imaging (MRI)-guided release.

## Introduction

Despite the tremendous advances in understanding the mechanisms driving tumor development and progression, cancer remains one of the major causes of worldwide deaths.^1^ Tumor diagnosis and tumor therapy are equally important for following tumor progression, providing targeted drug release, and applying effective treatment. Magnetic resonance imaging (MRI) is a noninvasive and widely used radiation-free tool with high-resolution anatomical gain.^2^ MRI works on the principle of nuclear magnetic resonance of protons,^3^ and contrast agents based on elements such as iron,^4,5^ gadolinium,^6^ or manganese^7^ are used in MRI to darken or brighten the proton image contrast. The quality of contrast agents is generally measured in terms of relaxivity (*r*_i_) rates: (1) longitudinal (*r*_1_), which is based on the spin–spin relaxation time, *T*_1_ and (2) transverse (*r*_2_), which is based on the spin–lattice relaxation time, *T*_2_. Iron oxide-based nanoparticles (IONPs) are FDA-approved *T*_2_ contrast agents,^8,9^ and they have the additional advantage of dissipating heat under alternating magnetic fields (AMFs),^10,11^ in a treatment called “magnetic hyperthermia (MH)”. This treatment was recently approved in Europe^11,12^ as a therapy to locally treat solid tumors at therapeutic temperature values above 43 °C. The high dosage of IONPs administered in clinical MH treatment does not permit the utilization of *T*_2_ contrast for imaging purposes. Moreover, due to the nondegradability of the used particles in MH, the whole tumor area tends to darken with no possibility of monitoring the tumor progression after MH treatments.^12−14^ Merging MRI with MH could overcome the decade-long disadvantage of MH itself in detecting optimal reservoirs of material doses at the target site to initiate treatment. Our group has recently developed iron oxide nanocubes (IONCs), which have benchmark performance in MH at clinically safe conditions, and are *in vivo* degradable at different rates depending on the type of coating,^15^ and act as *T*_2_ contrast agents when individually polymer-coated or encapsulated in polymer beads.^16,17^ To overcome their darkening contrast, we aimed to merge such nanoheaters with a positive contrast agent based on gadolinium (Gd).^3^ Indeed, positive contrast agents, like paramagnetic Gd^3+^-based chelates (Gd-DTPA/Gd-DOTA), have been used in the clinic as bright *T*_1_ contrast agents.^18,19^ Although FDA has approved the use of Gd^3+^ chelates, there have been reports linking their use to nephrotoxicity, attributed to the leakage of ions from the complex. Also, it was reported that *T*_1_ signals from some IONPs were (in values) higher than those from Gd chelates (4.4 mM^–1^·s^–1^), e.g., Feridex (10 mM^–1^·s^–1^)^20^ and cubic IONPs (39.3 mM^–1^·s^–1^),^16^ but their strong *T*_2_ signal attenuates the *T*_1_ signal, which is reflected by their higher *r*_2_/*r*_1_ ratio. In the past decade, research has shifted toward *T*_1_/*T*_2_ multimodal contrasting agents.^21−23^ Unlike monomodal MRI, *T*_1_/*T*_2_ dual-mode contrast imaging facilitates cross-validation of the target site with high precision, thus preventing false-positive results and artifacts, minimizing the risk of misinterpretation and erroneous diagnoses.^24−26^ Although IONPs were traditionally used for *T*_2_, in their ultrasmall sizes (below 10 nm),^27^ they were also exploited as *T*_1_-weighted contrast agents,^27,28^ but the idea of using such tiny IONPs for MH treatment was withdrawn due to their poor heating properties in the biological window of AMFs. Indeed, heating efficiency under an alternating magnetic field depends not only on nanoparticle features such as size, shape, and composition but also on AMF parameters such as frequency (*f*) and field amplitude (*H*). However, for the clinical use of MHT, AMF conditions are restricted to *H* × *f* factors below 5 × 10^9^ Am·s^–1^,^11^ with clinical trials done at 110 kHz and 24 kA m^–1^. For superparamagnetic nanoparticles, the smaller the particles are in size the less they heat because, according to the linear response theory, heating efficiency depends on the magnetic volume and saturation magnetization.^29^ It has been proven that iron oxide nanoparticles below 10 nm do not heat significantly while 15 nm iron oxide nanoparticles are able to heat; hence, we chose to work with iron oxide nanocubes of 15 nm in cube edge.^11^

In this report, we have designed a protocol to enwrap magneto-gadolinium-based nanostructures to combine an iron oxide nanocube suitably designed for magnetic hyperthermia with gadolinium fluoride NPs as a dual *T*_1_ MRI contrast agent. Recently, Li et al. reported a strategy for pairing *T*_1_/*T*_2_ dual imaging with a doxorubicin/hyperthermia combination therapy on a tannic acid-modified avocado-like Fe^3+^/Fe_2_O_3_ nanosystem for cancer theragnosis.^21^ Jiang et al. in another work reported the biosynthesis of Mn-doped magnetosomes, which enabled dual-mode *T*_1_/*T*_2_ imaging along with the possibility of photothermal therapy (PTT).^30^ However, both of these works differ in the adoption of a photoirradiation source for thermal therapy, rather than magnetic hyperthermia as done here. In another work, Chou et al. engineered FeGdPt alloys for dual *T*_1_/*T*_2_ and CT imaging.^31^ This work showed promising results as a multifunctional imaging probe in cells and mice; however, it did not have any therapeutic application. Moreover, the nanosystems mentioned above were designed for dual *T*_1_ and *T*_2_ contrast but lacked a tumor-selective contrast switching signal. In contrast, our system utilizes Gd-based NPs as a *T*_1_ contrast agent and by incorporating polymers sensitive to proteolytic cleavage and pH variation results in a theranostic platform responsive to tumor-sensitive stimuli. The methods adopted in the past to prepare *T*_1_/*T*_2_ nanostructures generally involved either direct doping,^32^ coencapsulation,^33,34^ or mesoporous silica loading.^24,25,35,36^ However, these methodologies have certain limitations, including the use of a massive amount of Gd-based chelates, which can raise concern for their toxicity^37^ or in the utilization of magnetic nanoparticles ineffective in providing suitable thermal doses for MH therapy.

Here, IONPs of cubic shape were synthesized via the thermal decomposition method^38^ and packed within an amphiphilic polymer to provide magnetic nanobeads (MNBs)^16,17^ as the base material for magnetic heating and MRI *T*_2_ imaging. NaGdF_4_ NPs) were selected as *T*_1_ enhancer because of their stable structure and bright contrast.^19,39^ NaGdF_4_ NPs coated with negatively charged poly(acrylic acid) were carefully loaded onto the MNB surface, mediated by an intermediate positively charged and an enzymatically cleavable poly-l-arginine hydrochloride (PARG) polymer by applying a layer-by-layer (LbL) approach.^40^ These magneto-gadolinium nanostructures provide a valuable temperature increase of nearly 9 °C, with significant specific absorption rate (SAR) values under biologically acceptable AMFs. Notably, the *T*_1_ signal of the NaGdF_4_ NPs exhibited a substantial reduction when densely packed on the bead surface, closely situated to the IONCs within the assembled nanostructure. Conversely, exposure to an intracellular protease enzyme solution led to recovery of the *T*_1_ signal, indicating PARG polymer cleavage. This process occurred over a 24 h incubation period at 37 °C, resulting in the disassembly of NaGdF_4_ NPs from the magnetic nanobeads (MNBs). This was readable by an increase in the *T*_1_ signal and the appearance of a bright signal in MRI. Moreover, these structures exhibited good cytocompatibility with the human glioblastoma U87 cell line and this portrayed them as excellent candidates for cancer theranostic strategies relying on a switchable contrast enhancer for MRI and MH therapy.

## Results and Discussion

**Figure 1:**
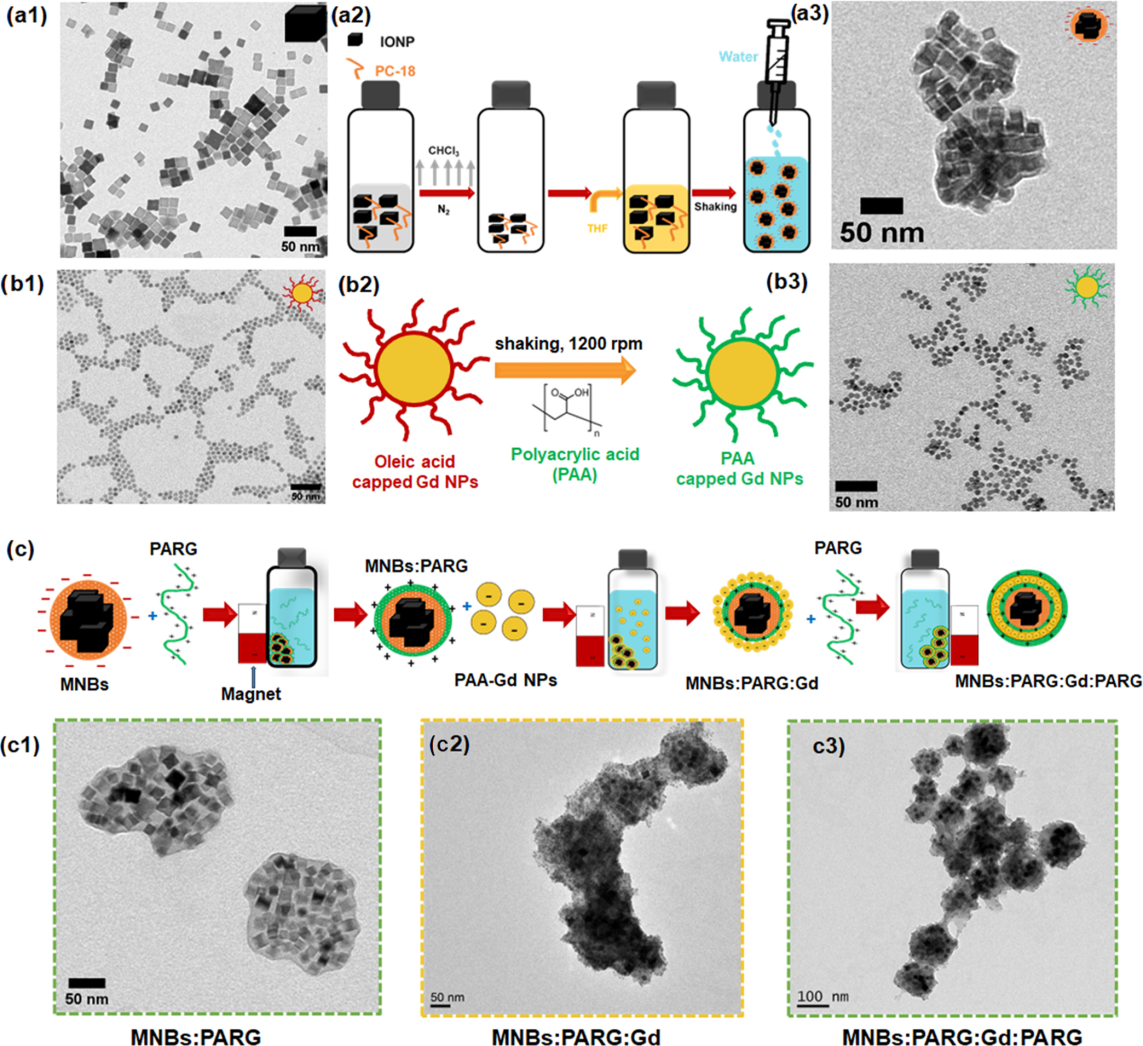
Preparation of enzyme-responsive magnetic-Gd multilayered nanostructures. (a1) TEM image of as-synthesized, IONCs of size 15 ± 2 nm used for the production of beads, (a2) schematic illustration of the microemulsion-based protocol to produce MNBs, and (a3) TEM image of corresponding water-soluble MNBs. (b1) As-synthesized hydrophobic oleic acid-capped Gd NPs, (b2) scheme depicting water-transferred hydrophobic Gd NPs using poly(acrylic acid), and (b3) TEM image of water-transferred Gd NPs after ligand exchange with PAA. (c) Schematic overview of assembling of PAA-Gd NPs and MNBs mediated by PARG layers. TEM images of (c1) MNBs after coating with the first layer of PARG (MNBs:PARG), (c2) after subsequent coating of MNBs:PARG with PAA-Gd NPs (MNBs:PARG:Gd), and (c3) after final coating of MNBs:PARG:Gd with a last layer of PARG (MNBs:PARG:Gd:PARG).

Multilayered magneto-gadolinium-based nanostructures for combining MH with dual MRI contrast were produced using an electrostatic-inspired layer-by-layer (LbL) method.^40^ For this, MNBs were first prepared according to a procedure previously reported by us and used as the core nanostructures for the next deposition of LbL.^17^ Hydrophobic IONCs of edge length 15 ± 2 nm (Figure 1a1) were chosen for the assembly in MNBs due to their low dependency of their heating efficiency in MH on the viscosity.^41^ Briefly, in a mixture of tetrahydrofuran (THF)/water, by an emulsion-based encapsulation approach, the nanocubes were wrapped within an amphiphilic polymer, poly(maleic anhydride-*alt*-1-octadecene) (PC18, Scheme in Figure 1a2; the details are in the Materials and Methods section). Each MNB is composed of multiple IONCs embedded in a thin layer of polymer as observed under a TEM (Figure 1a3 and additional images in Figure S1). MNBs exhibited a homogeneous water solution with a monomodal peak under dynamic light scattering (DLS), an average hydrodynamic size measured by an intensity of 193 nm, a full width at half-maximum (FWHM) of 88 nm, and a poly(dispersity index) (PDI) of 0.169 (Figure S2).

Next, for the preparation of NaGdF_4_ nanoparticles (Gd NPs) as *T*_1_ (positive/bright) contrast agents, hydrophobic oleic acid-coated Gd NPs (Figure 1b1) were prepared via thermal decomposition according to a published method (Figure S3),^19^ and water transferred by embedding each individual NP within a commercially available poly(acrylic acid) ligand (PAA, 1800 Da) using a well-established ligand-exchange protocol reported previously.^19^

As schematized in Figure 1b2, a CHCl_3_ mixture of Gd NPs and an excess of PAA polymer were dissolved in ethanol under sonication and shaken for 72 h at 1200 rpm (details are provided in the Methods section). The product was washed several times by precipitation with ethanol/hexane mixture (1:8 volume ratio) and was finally dissolved in Milli-Q water. The water-soluble PAA-coated Gd NPs (PAA-GdNPs) showed no sign of aggregation, with a monomodal DLS hydrodynamic peak with a maximum at 9 ± 1 nm (Figure S4), and no structural changes were observed in the TEM image (Figures 1b3 and S1) compared to Gd NPs before the PAA coating. Compositional elemental analysis of Gd by inductively coupled plasma (ICP) spectroscopy of the PAA-Gd NPs confirmed the initial composition. The choice of PAA ligand was solely made knowing its high hydrophilicity and water-retention capacities, the factors that crucially affect the *T*_1_ relaxation at the surface of Gd NPs. Indeed, any modification at the surface of the paramagnetic particles could largely impact their effective proton relaxation.^42,43^ Relaxivities of PAA-Gd NPs were compared with those of the same particles coated with an in-house synthesized catechol-based PEG molecule (TEM image in Figure S5) having a molecular weight similar to that of PAA and prepared by a ligand-exchange protocol.^44^ Here, the *r*_1_ relaxation capabilities of PAA-coated particles were 2-fold higher than the catechol-based PEG-coated ones (CAPEG-Gd NPs) at each of magnetic static field conditions measured (0.5, 1.0, and 1.5T, Figure S6). This was attributed to the high swelling and charge of the hydrophilic PAA coating.

MNBs and PAA-Gd NPs, now in water, were then assembled together into core–shell-like nanostructures using an LBL electrostatic approach,^40^ according to the procedure schematized in Figure 1c. MNBs and PAA-Gd NPs have negative surface charges of −50 and −21 mV, respectively, by ζ-potential measurements (Figure S7). The PARG polymer was used for the assembly. First, a presonicated aqueous dilution of MNBs (0.06 mg_Fe_ mL^–1^) was added dropwise into the PARG solution (0.5 mg mL^–1^ in 50 mM NaCl solution, pH 7) under sonication to prevent aggregation. After 1 h of shaking on an orbital shaker (1000 rpm), MNBs coated with PARG (MNBs:PARG) were magnetically recovered and dissolved in Milli-Q water. This process was repeated three times to remove free traces of the PARG polymer from the sample. The solution containing the extra free PARG polymer left after this first magnetic separation was preserved for reuse in the final wrapping of the composite. The magnetically recovered MNBs:PARG (0.06 mg_Fe_ mL^–1^), shown in Figure 1c1 (see more TEM images in Figure S8), were dispersed in 1 mL of fresh saline solution for the second layering with PAA-Gd NPs to provide the assembly named MNBs:PARG:Gd. To this aim, MNBs:PARG were added dropwise in a solution of PAA-Gd NPs (0.3 mg_Gd_ mL^–1^, in 50 mM NaCl solution, pH 7) under sonication and incubated for 3 h. Next, the reaction mixture was exposed to the magnet three times to finally recover the nanostructures (MNBs:PARG:Gd, Figure 1c2). The presence and distribution of Gd NPs on MNBs:PARG:Gd was confirmed clearly through structural characterization by TEM when comparing the initial MNBs with the MNBs:PARG:Gd at different stages of the layer-by-layer process (Figures 1c1–c3 and S8). Final wrapping of MNBs:PARG:Gd was accomplished by reusing the PARG-recovered solution in the first washing step to provide the final assembly coated by a PARG-positive layer named MNBs:PARG:Gd:PARG. The Gd composition in the final structure can be tuned by adjusting the concentration of PAA-Gd NPs used for the reaction. For instance, when a reaction was performed at 1 mg_Gd_ mL^–1^ PAA-Gd NPs, instead of 0.3 mg_Gd_ mL^–1^, as determined by ICP, it produced a final structure with an Fe:Gd ratio of 1:2.4 (mass ratio) rather than 1:0.8. The surface of MNBs:PARG:Gd structures (Figure 1c3), owing to the PARG coating (Figure S9), offered a soft surface appearance with more compact PAA-Gd NPs just at the surface of the MNBs. As demonstrated in Figure S10, these structures reversibly accumulate and redisperse in solutions after magnetic accumulation, with no effect on the quality of the materials. The observations that we imaged under TEM were complemented by the DLS data showing good agreement in terms of stability and quality of beads, even in solution, when considering the hydrated forms at each stage of functionalization (Figure S2).

**Figure 2:**
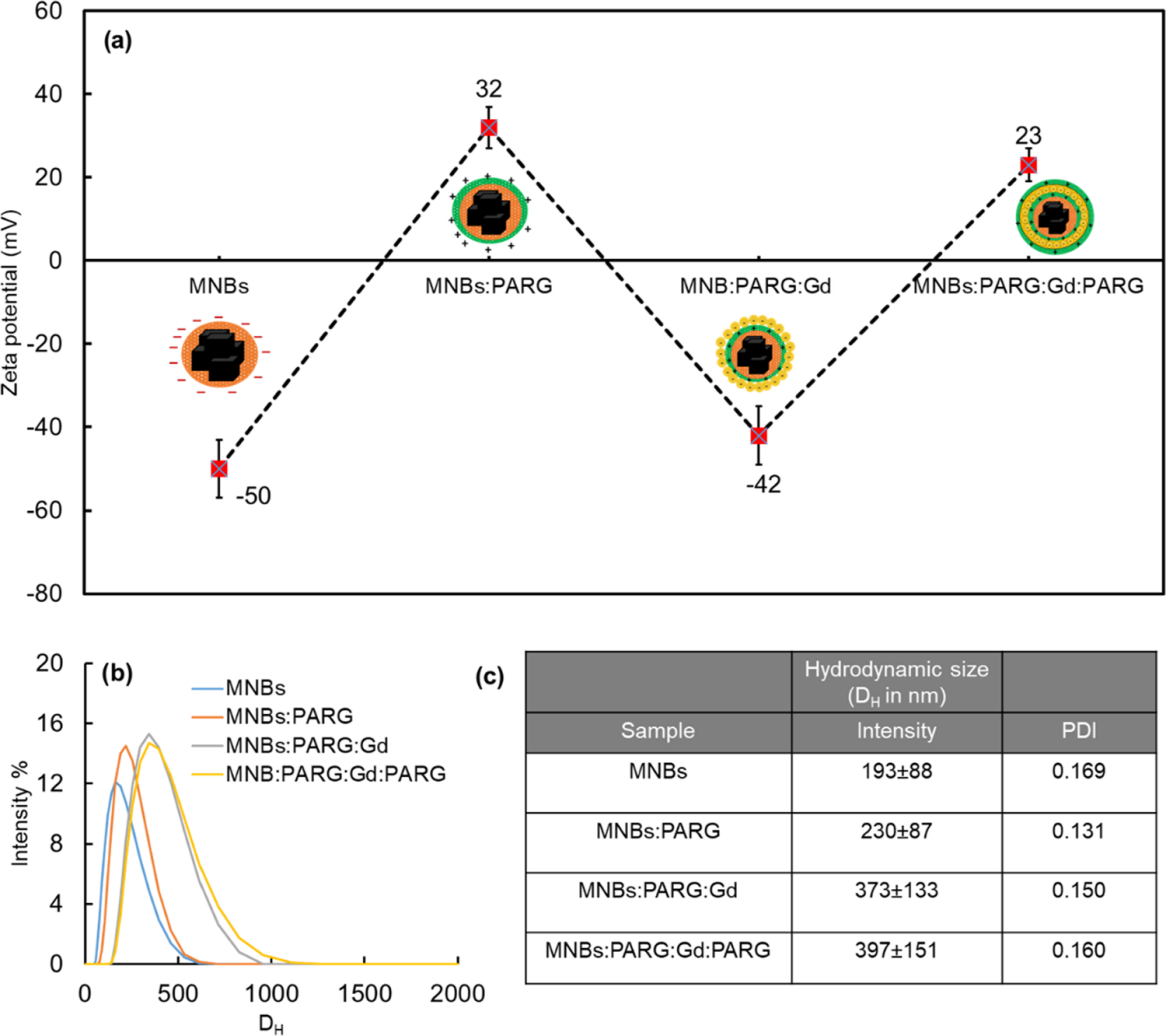
Surface ζ-potential and hydrodynamic diameter (*D*_H_) of magneto-gadolinium structures at each stage of layering. (a) ζ-Potential measurement of the magneto-gadolinium structure at each stage of layering at each assembly step. The positive charge of the MNBs:PARG and MNBs:PARG:Gd:PARG is attributed to the surface PARG layer, whereas for the MNBs:PARG:Gd, the negative charge is from the PAA-Gd NPs. (b) Comparison of the mean hydrodynamic size intensity (%) profiles for the structures at each stage of the layering process. (c) Summary table showing the mean values of hydrodynamic diameter reported with FWHM size distribution of the corresponding DLS peaks and PDI values.

The change of the surface charge was measured at each stage of layering by ζ-potential measurements, and the values were switched between negative and positive charges corresponding to the coating with the positive PARG layer or to negative value for the MNBs:PARG:Gd after assembling the Gd NPs on top of the MNB surface, as summarized in Figure 2a. For instance, the initial superficial charge of MNBs, i.e., −50 mV (Figure S7), shifted to +32 mV after the first PARG layering and back to −42 mV after embedding PAA-Gd NPs (initial charge of PAA-Gd NPs is −21 mV, Figure S7) on the beads for the product named MNBs:PARG:Gd, and finally, reversed to +25 mV after the next PARG embedding for the final product named MNBs:PARG:Gd:PARG. The layering process was further confirmed by the progressive increase in hydrodynamic size measured by DLS (Figure 2b), where the initial hydrodynamic size, 193 ± 88 nm (based on the intensity and reported as the mean size value ± full width at half-maximum) of MNBs, increased gradually up to 315 ± 119 nm after the layering process was finalized for the MNBs:PARG:Gd:PARG product (Figures 2b and S2). The polydispersity index (PDI) for these samples was also significantly low (<0.2), indicating a quite homogeneous sample in terms of distribution (Figure 2c).

**Figure 3:**
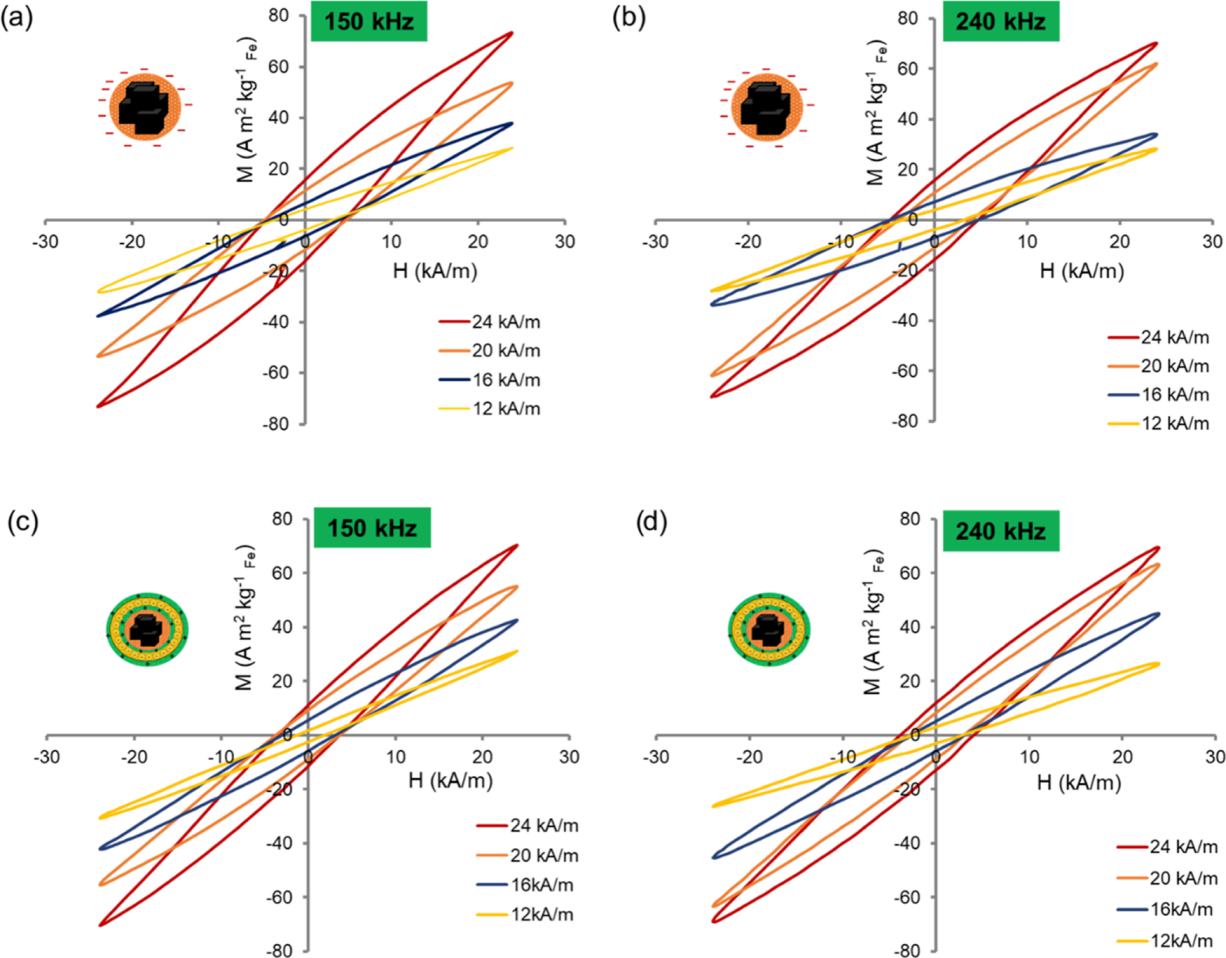
AC hysteresis loops. Hysteresis loops of MNBs (a,b) and of MNBs:PARG:Gd:PARG (c,d) at fixed frequencies of 150 or 240 kHz and in a magnetic field intensity range of 12–24 kA m^–1^, measured at a fixed iron dose of 1 mg_Fe_ mL^–1^ in 40 μL of water.

To evaluate the use of such structures in magnetic hyperthermia, the thermal losses of the different nanostructures were determined using dynamic magnetization measurements. AC hysteresis loops refer to energy losses of magnetic materials under alternating magnetic fields. An AC magnetometer was used to record the hysteresis loops of MNBs and MNBs:PARG:Gd:PARG in an aqueous solution (1 mg_Fe_ mL^–1^ in 40 μL) in response to time-varying magnetic fields at frequencies of 240 and 150 kHz and a field amplitude range of 12–24 kA m^–1^ (Figure 3). These magnetic field conditions were precisely selected to evaluate the SAR within the biological window of the AMFs, i.e., less than 5 × 10^9^ Am^–1^·s^–1^. The hysteresis loop area values (Table S1) and saturation magnetization values (Table S2) of MNBs after embedding were measurable for all samples. Values for MNBs:PARG:Gd:PARG were lower by an average of 10–20% (Figure 3c,d) than those of bare MNBs (Figure 3a,b) at all of the tested conditions.

**Figure 4:**
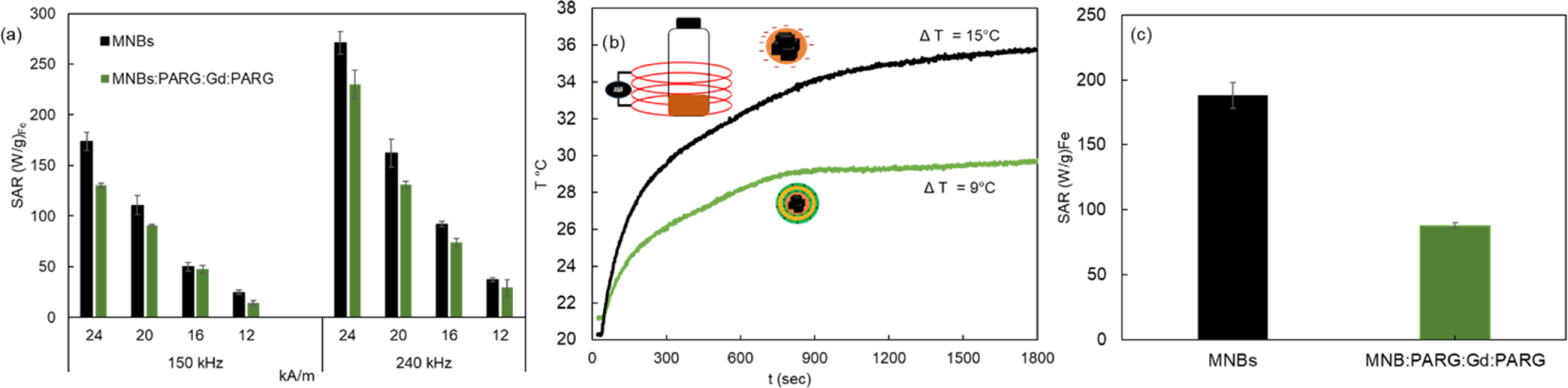
Magnetic hyperthermia properties: (a) Comparison of the SAR values of MNBs and MNB:PARG:Gd:PARG dissolved in aqueous solutions and measured by AC magnetometry to record the hysteresis loop areas at frequencies of 150 kHz and 240 kHz and a field range of 12–24 kA m^–1^ and applying eq 1. (b) Temperature versus time curves of the same samples after 30 min of continuous exposure to AMFs of 180 kHz and 30 kA m^–1^ using a calorimetric setup. Temperature differences (Δ*T*) for MNB and MNB:PARG:Gd:PARG samples recorded after 30 min under continous AMF exposure. (c) SAR values of MNBs and MNB:PARG:Gd:PARG, derived from calorimetry measurements using eq 2 (as described in the Methods section).

Based on the frequency and hysteresis loop areas measured, the corresponding SAR values of MNBs:PARG:Gd:PARG and MNBs were calculated according to eq 1 (see the Methods section). As expected, the SAR values of MNBs:PARG:Gd: PARG were always lower than those of the MNBs at both the frequencies of 240 and 150 kHz (Figure 4a). Moreover, these data were also confirmed by measuring the SAR using a calorimetric device: in this case, for MNBs and MNBs:PARG:Gd:PARG, the temperature profiles were recorded within 30 min of AMF exposure at 180 kHz and 30 kA m^–1^ (Figure 4b), providing a temperature difference (Δ*T*) of around 15 and 9 °C for the aqueous solution of MNBs and MNBs:PARG:Gd:PARG, respectively (Figure 4b). Notably, no significant temperature change was recorded for the water blank exposed under the same field conditions and for the same time (Figure S11). The average SAR derived from the calorimetry measurements using eq 2 (see the Methods section) was found to be 188 ± 10 W g_Fe_^–1^ for MNBs and 88 ± 2 W g_Fe_^–1^ for MNBs:PARG:Gd:PARG (Figure 4c).

The reduction in SAR values (Figure 4) measured by both methods on MNBs:PARG:Gd:PARG (Figure 2b) with respect to the SAR of MNBs may reflect the increase in the hydrodynamic size that apparently affects even more the Brownian relaxation mode of the nanostructures, thus reducing the loop area or the temperature profiles in agreement with previous findings.^11,17,45−47^

**Figure 5:**
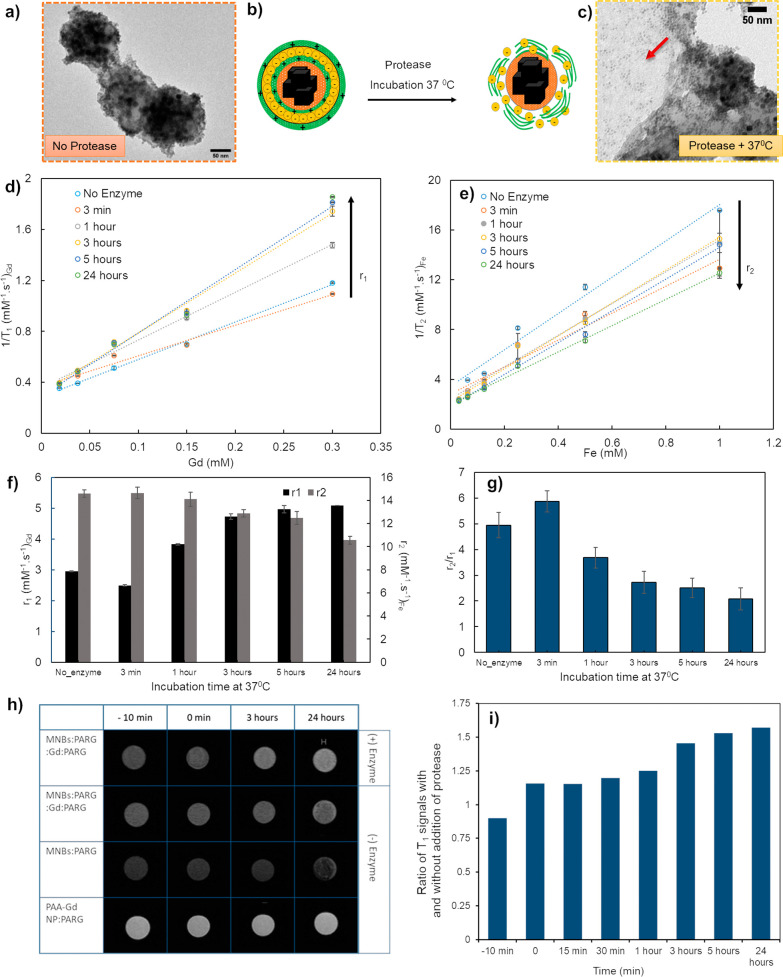
MRI relaxation properties. (a) TEM images of MNBs:PARG:Gd:PARG before enzyme treatment. (b) Schematic of the enzymatic degradation of nanostructures. (c) TEM images of MNBs:PARG:Gd:PARG after treatment with protease enzyme for an incubation time of 3 h. (d) Plot of 1/*T*_1_ values versus Gd concentration and (e) plot of 1/*T*_2_ values versus Fe concentration at different time points of incubation at 37 °C with protease enzyme for these MNBs:PARG:Gd:PARG. Data were fitted with linear fit and plotted as dotted lines. (f) *r*_1_ and *r*_2_ rates obtained as the slopes from the linear fitting of 1/*T*_1_ (d) and 1/*T*_2_ (e), and corresponding to (g) *r*_2_/*r*_1_ ratios plotted at different time points for MNBs:PARG:Gd:PARG with protease [data presented are average of three independent measurements (*n* = 3)]. (h) 3T MRI phantom images of MNBs:PARG:Gd:PARG in 0.15 wt % agarose solution at different incubation times. These images were compared with those of MNB-PARG and PAA-GdNP-PARG. (i) Histogram of the ratio of *T*_1_ values between MNBs:PARG:Gd:PARG with and without the addition of protease enzyme, as obtained from MRI at different time points.

Next, the MRI relaxivities of MNBs and PAA-GdNP samples in 0.5% agarose gels were used as a reference to estimate the signal of the building blocks before their assembly in the nanostructures. Under a static magnetic field of 1.5T, MNBs had relaxivities *r*_1_ = 1.5 mM^–1^ s^–1^, *r*_2_ = 269 mM^–1^ s^–1^, and a relaxivity *r*_2_/*r*_1_ ratio of 180, while those of PAA-GdNPs were *r*_1_ = 6 mM^–1^ s^–1^, *r*_2_ = 12 mM^–1^ s^–1^, and an *r*_2_/*r*_1_ ratio of 2 (Figures S12 and S13). The PAA-Gd NPs have an *r*_2_/*r*_1_ ratio close to 2, whereas MNBs with *r*_2_/*r*_1_ ratios much higher than 10 justify our selection of the material for their strong positive and negative signals, respectively.^3,48^ When assembled together in the final MNB:PARG:Gd:PARG (TEM in Figure 5a) nanostructure, the measured *r*_1_ and *r*_2_ values were found to be 2.9 and 14.5 mM^–1^ s^–1^, respectively, with an *r*_2_/*r*_1_ ratio of 4.9 (Figure 5d–g, no enzyme). The differences in relaxivities, from their individual forms, are likely due to the change in the hydrodynamic size of the assembly and exposure to water of the MRI contrast agents.

To monitor the changes in the relaxivity of MNB:PARG:Gd:PARG in response to intracellular protease enzymes, the relaxivity of MNB:PARG:Gd:PARG was assessed before enzyme exposure (no enzyme, Figure 5). Subsequently, this initial state was compared with the *r*_1_ relaxivity of MNB:PARG:Gd:PARG observed in agarose gel after incubation with the enzyme at 37 °C for varying durations up to 24 h (scheme in Figure 5b). In the presence of the enzyme, the linear slope of 1/*T*_1_ versus Gd concentration increased over time from 3 min to 1, 3, 5, and 24 h (Figure 5d and Table S3). This was also accompanied by a change in the *r*_1_ signal with the gradual enhancement of the *r*_1_ signals recorded from 3 min to 3 h and with an additional slight increase after 24 h of incubation. The initial *r*_1_ = 2.9 mM^–1^·s^–1^ in the packed structure (Figure 5f) increased up to 5.08 mM^–1^·s^–1^ upon incubation with the enzyme for 24 h (Figure 5f), where it saturated at a value of 4.9 mM^–1^·s^–1^ at 72 h (incubation point not displayed). On the contrary, the *r*_2_ values were reduced by 27% (from *T*_2_ = 14.5 to 10.5 mM^–1^·s^–1^) in the same incubation time window (Figure 5e,f). To confirm that this change in the *r*_1_ signal was assignable to the disassembly of the nanostructures with consequent exposure of Gd NPs to the environment, structural studies were carried out using TEM on the nanostructures before and after incubation with the enzyme (Figure 5a,c, and additional images in Figure S14). The dissociation of PAA-Gd NPs from the surface of MNBs was distinctly observable by the clear apperance of dark dot-like spots on the grid (Figure 5c). These dots corresponded to PAA-Gd NPs that dissociated from the surface of MNBs, and their presence became evident only after exposure to protease. This contrast was notable as there were no observable dots before exposure (Figure 5a), indicating that the beads initially had a compact structure with no freely dispersed nanoparticles on the grid. On the same gel, the signal of MNB:PARG:Gd:PARG in enzyme-free conditions was recorded, and, as expected, no change in relaxivity signals was observed after incubation at 37 °C from 0 to 24 h, indicating the structural stability of the MNB:PARG:Gd:PARG nanostructure in the enzyme-free environment (Figure S15). Indeed, the *r*_1_ (2.9 mM^–1^·s^–1^) and *r*_2_ values (14.5 mM^–1^·s^–1^) at 0 h of incubation were almost the same as those recorded at 24 h (*r*_1_ = 2.7 mM^–1^·s^–1^ and *r*_2_ = 14.9 mM^–1^·s^–1^) in the absence of the enzyme (Figure S15).

The *r*_2_/*r*_1_ ratio can also be considered as an ideal parameter to analyze the contrast changes.^3^ Here, in our case, the *r*_2_/*r*_1_ ratio in the presence of the enzyme reduced from 5.87 mM^–1^·s^–1^ at 3 min to 2.1 mM^–1^·s^–1^ at 24 h (Figure 5g). The *r*_2_/*r*_1_ value of 2 corresponds to the threshold range reported in the literature^3,49,50^ for considering a system as a *T*_1_ agent (dotted red line in Figure 5g), confirming the gradual increase of the *T*_1_ signal (coming from Gd nanoparticles) after the disassembly process occurred and which was initially attenuated by MNBs in the packed configuration.

To validate the relaxometry findings, MRI phantom images were collected with a 3T preclinical MRI machine (Figure 5h). The contrast of MNB:PARG:Gd:PARG with or without the addition of protease enzyme at different time points was compared with those of PAA-GdNP-PARG and MNB-PARG, which were used as control samples and were not exposed to the enzyme. The bright *T*_1_ contrast for the MNB:PARG:Gd:PARG sample subjected to protease treatment gradually increased over time (Figures 5h and S16). The effect of protease on the increase in contrast was quantified by dividing the *T*_1_ signal obtained on the MRI phantom for MNB:PARG:Gd:PARG with and without the enzyme at each time point (Figure 5i). This was done to mitigate minor signal discrepancies at the initial time points obtained due to experimental constraints such as slight variations in temperature to maintain the value at 37 °C needed during the measurements. In 1 h, there was a 1.2-fold increase in contrast, and this elevation progressed to 1.5 times after 3 h of protease treatment. By the end of this study, the signal intensity of the same sample closely resembled that of PAA-Gd NP:PARG, suggesting a nearly complete release of all Gd NPs from the assembled nanostructures, as also evidenced by TEM characterization (Figure S14).

**Figure 6:**
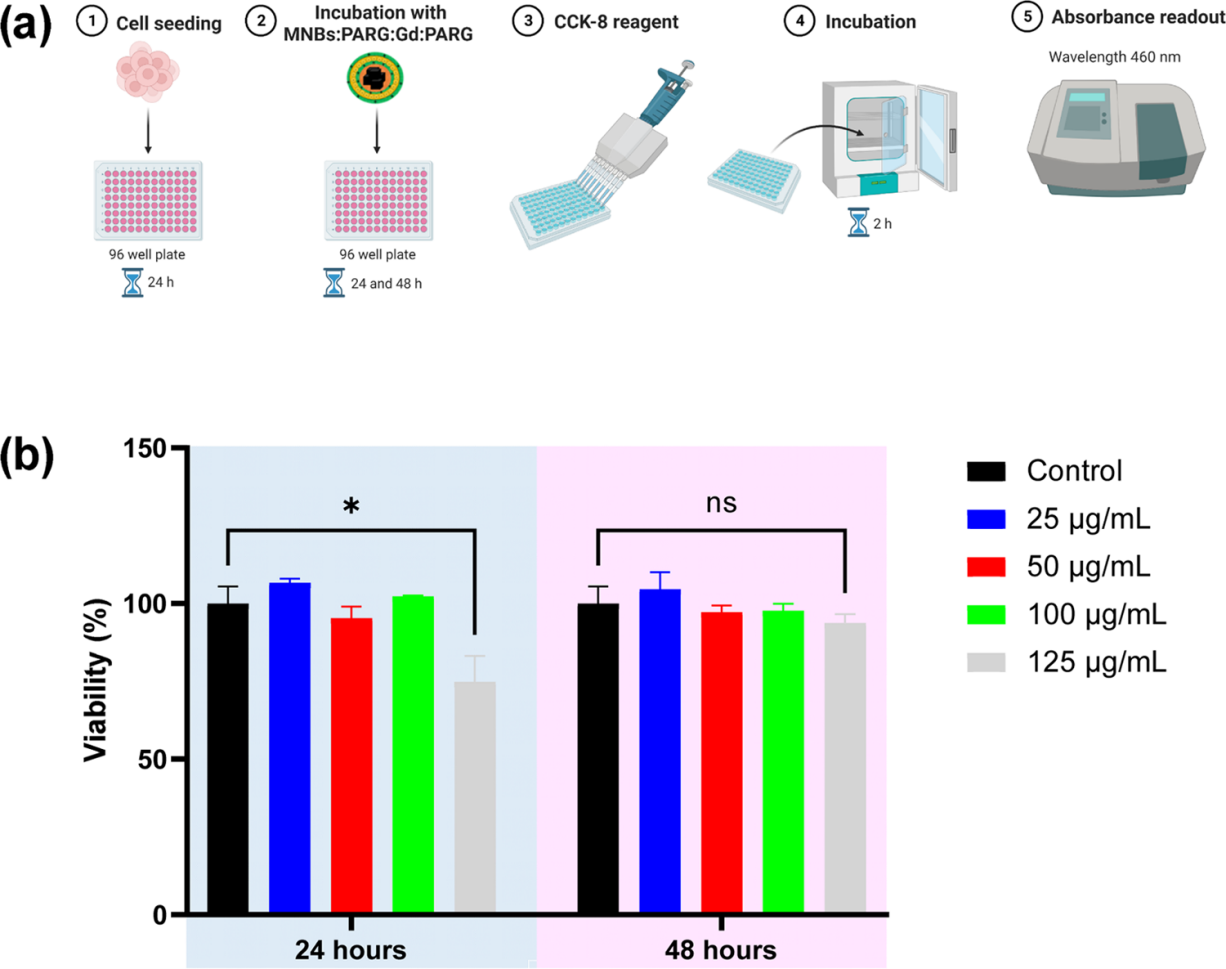
Cell toxicity evaluation of MNBs:PARG:Gd:PARG on U87 cells. (a) Schematic of the CCK-8 assay. Human glioblastoma U87 cells seeded in a 96-well plate were exposed for 24 and 48 h to MNBs:PARG:Gd:PARG with Gd and Fe content ranging from 25 to 125 μg mL^–1^ and from 10.4 to 52 μg mL^–1^, respectively. CCK-8 reagent was then added to each well and incubated at 37 °C for 2 h. After incubation, absorbance at 460 nm was measured using a plate reader. (b) Viability of U87 cells subjected to increasing doses of MNBs:PARG:Gd:PARG for 24 and 48 h. *n* = 3, mean ± standard error of the mean (SEM), *p < 0.033, two-way analysis of variance (ANOVA).

Potential toxicity is a primary concern in the use of nanomaterials in biomedical applications. Gadolinium dosage, in particular, due to nephrotoxicity concerns, is limited to 0.1 mmol kg^–1^ for injection in patients as an MR imaging contrast agent.^51^ To verify the relative safety of the proposed nanosystem, we assessed its cytotoxicity *in vitro* on a human glioblastoma U87 cell line by using a standard CCK-8 viability assay. U87 cells were exposed for 24 or 48 h to various amounts of the nanostructures, corresponding to a dose of gadolinium ranging between 25 μg mL^–1^ (2.5 μg Gd) and 125 μg mL^–1^ (12.5 μg Gd) and to a corresponding iron dose ranging from 10.4 μg/mL (1.04 μg Fe) to 52 μg mL^–1^ (5.2 μg Fe) (Figure 6a). The nanostructure had a safe profile, with a viability above 95% up to a Gd dose of 100 μg mL^–1^. A significant reduction in metabolic and proliferative activity was observed at the highest dose of Gd tested (125 μg mL^–1^) at 24 h, with cell viability that was rescued to control levels after 48 h (Figure 6b). No significant cytotoxic effects were induced in all of the other conditions. For an average human patient weighing 70 kg, the safe limit of Gd exposure is 1100 mg Gd; for 20 g mice, this value corresponds to 360 μg.^51^ Considering these values, our results demonstrate that our platform possesses an excellent toxicity profile and can be potentially applied for bioimaging and therapeutic applications.

Similar findings were reported by Ling et al., who prepared multimodal structures composed of iron oxide nanoparticles, phototherapeutic agents, and fluorescent agents with *T*_1_ signals that exhibited an acidic pH-responsive behavior. Indeed, even in these assemblies, the *T*_1_ signals that initially quenched at pH 7.7 were recovered after disassembling in an acidic solution (pH 5.5), which mimicked the tumor pH.^52^ In another study, a similar *T*_1_ enhancement strategy was exploited in a system composed of mesoporous silica-coated manganese oxide (MNO) nanoparticles modified with tumor-specific hyaluronic acid (HA), and the HA was digested using hyaluronidase enzyme with water access through the silica channels to the MNO core.^53^ However, the relative poor *r*_1_ values of 1.29 mM^–1^·s^–1^, which is 4 times lower than the values of our system, and the absence of auxiliary-integrated therapeutic properties represents a disadvantage with respect to our system. Among other studies of stimuli-responsive theranostic models,^54−57^ a recent study successfully integrated esterase-specific modulation of *T*_2_ relaxivity with the imaging of MPI signals from other nanostructures.^17,58^ Our nanosystem, developed here, stands out for the unique combination of high *r*_1_ values responsive to the tumor microenvironment to guarantee *T*_1_ monitoring signals for magnetic tracking and delivery and offers an *ad hoc* system designed for MH application for clinical use. The platform developed in this study demonstrated a significant improvement in the *T*_1_ contrast upon enzyme activation in phantoms. However, the substantial size of the structures and absence of targeting units on the surface may present a notable obstacle for systemic *in vivo* delivery. Addressing this challenge is crucial when aiming to apply this work to *T*_1_ contrast-guided MHT actuation in preclinical and clinical settings.

## Conclusions

This study presents a successful approach for crafting protease-responsive multilayered magnetic gadolinium iron oxide nanostructures. These nanostructures demonstrate effectiveness in both MH treatment and *T*_1_–*T*_2_ MRI dual imaging. Significantly, these assemblies provide the capability to selectively trigger *T*_1_ contrast in environments mimicking protease-rich intracellular conditions, thereby indicating the timing of nanostructures reaching tumor target release. Water-soluble MNBs as a core material for MH and *T*_2_ imaging were first produced by assembling iron oxide nanocubes designed for MH within an amphiphilic polymer, whereas the PAA-coated Gd NPs were employed as positive contrast agents (*T*_1_), decorating the MNB surface through a robust layer-by-layer process using a protease-cleavable PARG polymer. The respective *T*_2_ contrasting abilities and efficient heat-mediating properties of the nanostructure were preserved with significant SAR values under clinical conditions. While the *T*_1_ MRI signal of the assembled PAA-Gd NPs in the MNB:PARG:Gd:PARG assembly was initially hindered within the assembly with MNBs and their *T*_2_ signals, the exposure to protease enzyme led to the depletion of PARG layers with consequent dissociation of PAA-Gd NPs from the assembly, resulting in the recovery of *T*_1_ signals by up to 75% compared to the initial assembled and untreated samples. In addition, MNB:PARG:Gd:PARG did not show toxicity in the human glioblastoma U87 cell line up to maximum concentrations of 125 μg_Gd_ mL^–1^ and 52 μg_Fe_ mL^–1^. Such nontoxic and smart responsive nanostructures to environmental stimulations,^59^ such as acidic pH,^26,33,52^ enzymatic, or temperature,^60^ are rather interesting for real-time delivery studies. The promising results achieved with our assembled platform and their peculiar features based on the nanoparticle building blocks call for future research to validate these properties in animal models. In the next phase, we will aim at proving, *in vivo*, the precise localization of the assembly material to then activate, on-demand with spatial and temporal control, and to finally apply MH treatment at the tumor site.

## Materials

The following chemicals were used: gadolinium(III) oxide (Gd_2_O_3_), oleic acid (90%), trifluoroacetic acid (TFA), sodium trifluoroacetate, oleylamine (98%), tetrahydrofuran (99%), poly(maleic anhydride-*alt*-1-octadecene) (PC18, Mn 30,000–50,000, Aldrich), Milli-Q water (18.2 MΩ, filtered with a filter pore size of 0.22 μM) from Millipore, chloroform (CHCl_3_, Sigma-Aldrich, 99%), poly(acrylic acid) (PAA, Mn 1800 Da), 1-octadecene (1-ODE, 99%), poly-l-arginine hydrochloride (PARG, *M*_w_ > 70 kDa, no. P3892), and protease (no. P5147). All chemicals were purchased from Sigma-Aldrich and were used without any further purification. Iron(III) acetylacetonate (Acros Organics, 99%), decanoic acid (Acros Organics, 99%), dibenzyl ether (Acros Organic, 99%), and squalene (Alfa Aesar, 98%) were used.

## Methods

### Synthesis of Iron Oxide Nanocubes

Iron oxide nanocubes of edge length 15 ± 2 nm were synthesized via thermal decomposition, according to previously published methods.^17,61^ For this, 1 mmol (0.353 g) of iron(III) acetylacetonate, 4.5 mmol (0.76 g) of decanoic acid, 9 mL of squalane, and 16 mL of dibenzyl ether were dissolved and degassed for 120 min at 65 °C in a 100 mL three-neck flask. The mixture was heated to 200 °C at a rate of 3 °C min^–1^ and maintained at this temperature for 2 h. Later, the reaction temperature was increased further to 310 °C at a rate of 7 °C min^–1^, and continued for another 1 h. Finally, after cooling the solution to room temperature (RT), the particles were precipitated through centrifugation (4500 rpm) by adding 60 mL of acetone. This step was repeated twice, and at the last centrifugation, the dark pellet of particles was redispersed in 15 mL of chloroform.

### Synthesis of Sodium Gadolinium Fluoride Nanoparticles

Inorganic sodium gadolinium fluoride nanoparticles (Gd NPs) of size 7.5 ± 0.7 nm were synthesized by modifying the previously published thermal decomposition protocol.^19^ The precursor Gd^3+^ trifluoracetate (Gd^3+^TFA), for the Gd NP reaction, was prepared a day before the synthesis: in a 100 mL three-neck round-bottom glass flask, 0.75 mmol of Gd_2_O_3_ in 6 mL trifluoroacetic acid (TFA) and Milli-Q water (in 1:1 volume ratio) were mixed under magnetic stirring and reflux at 90 °C until a transparent solution was obtained. After overnight drying at 60 °C, the powder obtained was used as a Gd^3+^ precursor. Next, the synthesis of nanoparticles was initiated by adding the whole amount of Gd^3+^ precursor into the flask, 1.5 mmol of sodium trifluoroacetate (NaTFA), 5.3 mL of oleic acid, 10.7 mL of 1-octadecene, and 7.05 mL of oleylamine. Next, in a gentle manner, a vacuum was applied under magnetic stirring (1200 rpm), and the solution was heated at 100 °C and incubated at 100 °C for 30 min. When the solution appeared yellowish after complete dissolution of the precursors, the vacuum was replaced by a gentle flow of nitrogen, and the reaction temperature was ramped up to 310 °C at a rate of 10 °C min^–1^ and maintained at 310 °C for 10 min. Next, the solution was cooled to room temperature by removing the heating mantle. After adding 20 mL of ethanol, it was centrifuged at 5000 rpm to precipitate the particle fraction. The recovered pellet was redissolved in 10 mL of hexane, and the washing step was repeated three times, as described above. The final pellet of oleate-capped Gd NPs was dissolved and stored in 20 mL of CHCl_3_ for further use.

### Water Transfer of Sodium Gadolinium Fluoride Nanoparticles Using Poly(acrylic acid)

The hydrophobic Gd NPs were transferred into water with poly(acrylic acid) (PAA) using a slightly modified ligand exchange protocol, as reported previously.^19^ Briefly, in a 40 mL glass vial, 1 mL of Gd NPs (7.89 g_Gd_ L^–1^ in CHCl_3_) was dissolved under sonication in 8 mL of PAA ethanol solution (72 mg of PAA dissolved in 6 mL of ethanol). The reaction mixture was shaken at 1200 rpm on an orbital shaker for 72 h at room temperature. Next, the particles were precipitated by adding hexane (1:8 in EtOH/hexane volume ratio) and centrifuged at 6000 rpm for 20 min. The supernatant was discarded to remove the excess free polymer. Gd NPs were redissolved in 2 mL EtOH and the centrifugation step was repeated 3 times to ensure the complete removal of excess polymer. The GdNPs coated with PAA (PAA-GdNPs) were finally resuspended in 2 mL Milli-Q water and filtered through 1.2 μM hydrophilic syringe filters (Merck Millipore). As determined by ICP, the water transfer yield was around 93%.

### Synthesis of Magnetic Nanobeads

Magnetic nanobeads (MNBs) were prepared according to our previously reported procedure with slight modification.^16,17^ Briefly, to 20 μL of IONCs (2.14 g_Fe_ L^–1^ in CHCl_3_), 33 μL of 50 mM poly(maleic anhydride-*alt*-1-octadecene, PC18) in monomer units in CHCl_3_ was added and sonicated in a 8 mL glass vial for 1 min at room temperature (RT). After complete evaporation of the initial solvent (using a nitrogen flux), 200 μL of fresh THF was added, and the mixture was sonicated for an additional 2 min. The THF mixture was gradually destabilized by dropwise addition of 1.6 mL of Milli-Q water with a syringe pump at a flow rate of 2 mL min^–1^ on an orbital shaker set at 1250 rpm. To obtain the desired quantity of MNBs, this protocol was repeated 40 times. All sample products were merged and collected on a magnet (0.3T), redissolved in 10 mL of Milli-Q water, filtered (1.2 μM hydrophilic syringe filter), and concentrated to a final volume of 500 μL using a permanent magnet of 0.3T.

### Layer-by-Layer Process of Loading Gadolinium NPs on Magnetic Beads

The desired magnetic-Gd NP layered structure was prepared by combining MNBs and PAA-coated Gd NPs via a layer-by-layer (LBL) procedure inspired by previous work.^40^ In an 8 mL glass vial, 1 mL of aqueous suspension of MNBs (0.06 mg_Fe_ mL^–1^ in water) was injected dropwise under sonication into 1 mL of poly-l-arginine hydrochloride polymer solution (PARG, 0.5 mg mL^–1^ in 50 mM NaCl solution, pH 7.2). After incubating the mixture for 1 h at RT on an orbital shaker (1250 rpm), the PARG-coated beads (MNBs:PARG) were collected and separated from the free polymer in solution by means of a permanent magnet (0.3T). The MNBs:PARG were magnetically washed thrice with Mill-Q water and each time dispersed in 1 mL of fresh Milli-Q water. As a second layer, 1 mL of aqueous dispersion of PAA-Gd NPs (0.5 mg_Gd_ mL^–1^ in water) was added dropwise into 1 mL of solution of MNBs:PARG under sonication (1 min) and subsequently incubated for an additional hour on an orbital shaker at 1000 rpm. The MNBs:PARG structure layered with Gd NPs (MNB:PARG:Gd) was extracted, washed again using a magnet, and prepared for final layering by dissolving the pellet in 1 mL of fresh Milli-Q water. The final enwrapping of MNB:PARG:Gd in the PARG was performed by reusing the PARG supernatant preserved after the first layering. After repeating magnetic washings, the final product MNB:PARG:Gd:PARG was dissolved in 500 μL of Milli-Q water and stored for further investigation.

### Quantification of Iron and Gadolinium

The iron and gadolinium in solutions were quantified using an inductively coupled plasma atomic emission spectrometer (ICP-OES, iCAP 6500, Thermo). Prior to measurements, 10 μL of samples were digested overnight in 1 mL of aqua regia, diluted to 10 mL with water, and filtered (0.45 μM PTFE filter) before being submitted for analysis.

### Dynamic Light Scattering (DLS) and ζ-Potential Measurements

The hydrodynamic sizes (*d*_H_) of the nanostructures in aqueous media were determined by dynamic light scattering (DLS) using a Zetasizer (Nano ZS90, Malvern, U.K.), equipped with a 633 nm He–Ne laser (4.0 mW) and a photodiode detector. The *d*_H_ measurements were repeated three times on an average for each sample. Data are indicated with a distribution range obtained from the full width at half-maximum of the DLS peak. The samples for analysis were prepared by diluting 10–20 μL of the samples in 1 mL of Milli-Q water.

### Transmission Electron Microscopy (TEM) Images

The structure and morphology of nanostructures and building blocks were imaged using transmission electron microscopy (TEMJEOL, JEM-1011) at an accelerating voltage of 100 kV. Imaging samples were prepared by dropping diluted aqueous solutions of the samples on a carbon-coated copper grid and drying overnight at room temperature before imaging.

### AC Magnetometer Measurements

Hysteresis loops of samples were determined on 40 μL aqueous solutions of samples (at a fixed iron concentration of 1 mg_Fe_ mL^–1^) using an AC magnetometer (AC Hyster advance, Nanotech solutions).^17,62^ Magnetization curves (hysteresis loops) were measured at frequencies of 240 and 150 kHz and at varied fields (16, 20, and 24 kA m^–1^). The average of the curve area (*A*) from hysteresis loops (from three consequent measurements) multiplied by the frequency (*f*) and normalized to the iron dose (g·L^–1^) according to eq 1 were used to determine the specific absorption rates (SAR) as follows^63,64^
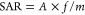
1

where *A* is the hysteresis area, *f* is the applied AC magnetic field frequency, and *m* is the iron weight in the dispersing medium volume.

### Calorimetric SAR Measurements

The SAR values of water-dissolved MNBs:PARG:Gd:PARG were also evaluated using a calorimetric magnetic hyperthermia device (DM 100 Series; nanoScale Biomagnetics Corp) and compared with MNBs (prior to the layering procedure). The analysis was performed at a fixed field and a frequency of 180 kHz and 30 kA m^–1^, respectively, within the biologically acceptable limit. For analysis, 180 μL of a finely sonicated suspension of the structure, at an iron concentration of 2.1 g L^–1^, was introduced into a 200 μL sample holder. The sample was inserted into the induction coil and exposed to alternating magnetic fields at a frequency of 180 kHz and a field amplitude of 30 kA m^–1^. The temperature increase of the solutions was recorded using an optic fiber thermosensor (LumaSense). The heating properties in terms of SAR were calculated as an average of three measurements, according to the formula given in previous reports.^11,17^
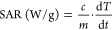
2where *C* is the heat capacity of water (4185 J L^–1^·K^–1^), *m* is the iron concentration in the solution (g L^–1^), and d*T*/d*t* is the initial slope value of the temperature increase in the first second time.

### Relaxivity Measurements

Longitudinal (*r*_1_) and transverse (*r*_2_) relaxation times of MNBs, PAA-GdNPs, and MNBs:PARG:Gd:PARG were measured using a Minispec spectrometer (Bruker, Germany) mq 60 (1.5T).^58^ In order to obtain first the relaxation times (*T*_1_ and *T*_2_), the calibration curves of 7 dilution points were prepared at a volume of 500 μL each in 0.5% agarose aqueous solution (wt %); the concentrations of iron ranged from 0.06 to 1 mM and those of gadolinium ranged from 0.01 to 1 mM. Relaxivities (*r*_*i*_, *i* = 1,2) were obtained from the slope of the linear intercept plot of relaxation rates (1/*T*_*i*_, *i* = 1,2) plotted against the concentration of Gd or Fe,^43^ which were the elements (elemental Gd for *r*_1_ and elemental Fe for *r*_2_) in our case. The data are presented as an average of three measurements (*n* = 3).
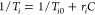
3Here, *T*_*i*_ represents the absolute relaxation time, *T*_*i*0_ is the relaxation time without an contrast agent, and *C* represents the concentration of an element (in mM).

The relaxation time values (in ms) obtained for calibration plotting are an average of three consecutive measurements. The relaxation times of MNBs:PARG:Gd:PARG were measured before and after adding 10 μL of protease (1.6 mg mL^–1^ in tris borate buffer, pH 7.6) to each dilution point (7 in total) and incubated at 37 °C. The measurements after protease treatment were performed at different time intervals of incubation (3 min, 1, 3, 5, and 24 h).

### MRI Contrast Imaging

In a PCR tube, the samples for MR imaging were prepared containing 1.9 μg of Gd and 2.4 μg of Fe, with a final volume of 200 μL in 0.15% agarose. The tubes were held in a custom-made holder in a specified sequence, as shown in Figure S14a. MR phantom studies were performed using a 3T small-animal MRI scanner (MRS*PET 7.0T, MR Solutions, Guildford, U.K.). The MR images were obtained using *T*_1_-weighted Fast Spin Echo (FSE-*T*_1_) sequence with the following parameters: echo time (TE) = 11 ms, repetition time (TR) = 1000 ms, field of view (FOV) = 50 × 50 mm, flip angle = 90°, and slice thickness = 1 mm. After 10 min of the acquisition of the first image, in one of the tube containing MNB:PARG:Gd:PARG, 10 μL of protease (1.6 mg mL^–1^ in tris borate buffer, pH 7.6) was added and this time was considered as *t* = 0 min for subsequent imaging studies. A steady temperature of 37 °C was maintained throughout the 24 h of the experiment by incubating the vials on a shaker at 37 °C when they were not being imaged.

### Cell Culture

All of the cell culture chemicals were purchased from Gibco. The U87 M glioblastoma cell line was acquired from ATCC. The cells were cultured in Dulbecco’s modified Eagle’s medium (DMEM, high glucose), supplemented with 10% fetal bovine serum (heat-inactivated FBS), 2% penicillin–streptomycin (10,000 U mL^–1^), and 1% l-glutamine (200 mM) at 37 °C, 5% CO_2_, and 95% relative humidity.

### Cytotoxic Cell Studies

The cytotoxic profile of MNB:PARG:Gd:PARG was analyzed using the CCK-8 assay. U87 cells were seeded in a 96-well plate (5 × 10^3^ cells per well) and allowed to grow for 24 h before incubation with MNB:PARG:Gd:PARG in 100 μL medium at concentrations ranging from 25 to 125 μg_Gd_ mL^–1^ and 10.4 to 52 μg_Fe_ mL^–1^. After incubation for 24 and 48 h, cell viability was measured using the CCK-8 assay according to the manufacturer’s instructions. Briefly, 10 μL of the CCK-8 reagent was added into each well and incubated for 2 h in an incubator under standard conditions (37 °C, 5% CO_2_, and 95% humidity). Then, absorbance at the wavelength of 460 nm was measured using a plate reader.

## Supporting Information Available

Additional TEM images of MNBs and PAA-coated Gd NPs; DLS peaks of MNBs and the MNB:PARG:Gd:PARG nanostructure at each stage of layering; DLS peaks of PAA-Gd NPs; TEM images of catechol PEG-coated Gd NPs and PAA-Gd NPs layered with PARG. *r*_1_, *r*_2_, and *r*_2_/*r*_1_ histograms of cathacol PEG-coated Gd NPs compared to PAA-Gd NPs; surface ζ-potential plots of MNBs and PAA-Gd NPs; additional TEM images of MNBs before layering; and MNBs:PARG, MNBs:PARG:Gd, and MNBs:PARG:Gd:PARG. Images demonstrating the reversible magnetic accumulation of MNBs:PARG:Gd:PARG; AC hysteresis loop areas of MNBs and MNBs:PARG:Gd:PARG; table of saturation magnetization values obtained from the AC hysteresis loop; linear calibration curves of MNBs and PAA-Gd NPs to determine the relaxivity; *r*_1_, *r*_2_, and *r*_2_/*r*_1_ histograms of MNBs and PAA-Gd NPs before and after conjugation with PARG; *r*_1_, *r*_2_, and *r*_2_/*r*_2_ histograms of MNBs:PARG:Gd:PARG without the enzyme incubated at 37 °C for 24 h; TEM images of MNB:PARG:Gd:PARG at different time points of incubation with protease enzyme; temperature versus time curve of water under alternating magnetic fields; *R*^2^ correlation coefficient values for the linear fitting of 1/*T*_1_ (Figure 5d) and 1/*T*_2_ (Figure 5e) of MNBs:PARG:Gd:PARG at different time points of enzyme incubation; MRI phantom contrast imaging of MNBs:PARG:Gd:PARG at different time points of enzyme incubation (PDF)

## References

[ref1] Organization, W. H. O. Cancer, 2023. https://www.who.int/news-room/fact-sheets/detail/cancer.

[ref2] Grover V. P.; Tognarelli J. M.; Crossey M. M.; Cox I. J.; Taylor-Robinson S. D.; McPhail M. J. Magnetic Resonance Imaging: Principles and Techniques: Lessons for Clinicians. J. Clin. Exp. Hepatol. 2015, 5 (3), 246–255. 10.1016/j.jceh.2015.08.001. 26628842 PMC4632105

[ref3] Lee N.; Yoo D.; Ling D.; Cho M. H.; Hyeon T.; Cheon J. Iron Oxide Based Nanoparticles for Multimodal Imaging and Magnetoresponsive Therapy. Chem. Rev. 2015, 115 (19), 10637–10689. 10.1021/acs.chemrev.5b00112. 26250431

[ref4] Fernández-Barahona I.; Muñoz-Hernando M.; Ruiz-Cabello J.; Herranz F.; Pellico J. Iron Oxide Nanoparticles: An Alternative for Positive Contrast in Magnetic Resonance Imaging. Inorganics 2020, 8 (4), 28 10.3390/inorganics8040028.

[ref5] Zhao S.; Yu X.; Qian Y.; Chen W.; Shen J. Multifunctional Magnetic Iron Oxide Nanoparticles: An Advanced Platform for Cancer Theranostics. Theranostics 2020, 10 (14), 6278 10.7150/thno.42564. 32483453 PMC7255022

[ref6] Kim H.-K.; Lee G. H.; Chang Y. Gadolinium as an Mri Contrast Agent. Future Med. Chem. 2018, 10 (6), 639–661. 10.4155/fmc-2017-0215. 29412006

[ref7] Cai X.; Zhu Q.; Zeng Y.; Zeng Q.; Chen X.; Zhan Y. Manganese Oxide Nanoparticles as Mri Contrast Agents in Tumor Multimodal Imaging and Therapy. Int. J. Nanomed. 2019, 14, 8321 10.2147/IJN.S218085. PMC6814316 31695370

[ref8] Fatima H.; Kim K.-S. Iron-Based Magnetic Nanoparticles for Magnetic Resonance Imaging. Adv. Powder Technol. 2018, 29 (11), 2678–2685. 10.1016/j.apt.2018.07.017.

[ref9] Issa B.; Obaidat I. M. Magnetic Nanoparticles as Mri Contrast Agents. Magn. Reson. Imaging 2019, 378, 40.

[ref10] Fortin J.-P.; Wilhelm C.; Servais J.; Ménager C.; Bacri J.-C.; Gazeau F. Size-Sorted Anionic Iron Oxide Nanomagnets as Colloidal Mediators for Magnetic Hyperthermia. J. Am. Chem. Soc. 2007, 129 (9), 2628–2635. 10.1021/ja067457e. 17266310

[ref11] Gavilán H.; Avugadda S. K.; Fernández-Cabada T.; Soni N.; Cassani M.; Mai B. T.; Chantrell R.; Pellegrino T. Magnetic Nanoparticles and Clusters for Magnetic Hyperthermia: Optimizing Their Heat Performance and Developing Combinatorial Therapies to Tackle Cancer. Chem. Soc. Rev. 2021, 50 (20), 11614–11667. 10.1039/D1CS00427A. 34661212

[ref12] Thiesen B.; Jordan A. Clinical Applications of Magnetic Nanoparticles for Hyperthermia. Int. J. Hyperthermia 2008, 24 (6), 467–474. 10.1080/02656730802104757. 18608593

[ref13] Hilger I. In Vivo Applications of Magnetic Nanoparticle Hyperthermia. Int. J. Hyperthermia 2013, 29 (8), 828–834. 10.3109/02656736.2013.832815. 24219800

[ref14] Hilger I.; Kaiser W. A. Iron Oxide-Based Nanostructures for Mri and Magnetic Hyperthermia. Nanomedicine 2012, 7 (9), 1443–1459. 10.2217/nnm.12.112. 22994960

[ref15] Mai B. T.; Balakrishnan P. B.; Barthel M. J.; Piccardi F.; Niculaes D.; Marinaro F.; Fernandes S.; Curcio A.; Kakwere H.; Autret G.; et al. Thermoresponsive Iron Oxide Nanocubes for an Effective Clinical Translation of Magnetic Hyperthermia and Heat-Mediated Chemotherapy. ACS Appl. Mater. Interfaces 2019, 11 (6), 5727–5739. 10.1021/acsami.8b16226. 30624889 PMC6376448

[ref16] Materia M. E.; Guardia P.; Sathya A.; Pernia Leal M.; Marotta R.; Di Corato R.; Pellegrino T. Mesoscale Assemblies of Iron Oxide Nanocubes as Heat Mediators and Image Contrast Agents. Langmuir 2015, 31 (2), 808–816. 10.1021/la503930s. 25569814

[ref17] Avugadda S. K.; Materia M. E.; Nigmatullin R.; Cabrera D.; Marotta R.; Cabada T. F.; Marcello E.; Nitti S.; Artés-Ibañez E. J.; Basnett P.; et al. Esterase-Cleavable 2d Assemblies of Magnetic Iron Oxide Nanocubes: Exploiting Enzymatic Polymer Disassembling to Improve Magnetic Hyperthermia Heat Losses. Chem. Mater. 2019, 31 (15), 5450–5463. 10.1021/acs.chemmater.9b00728. 31631940 PMC6795213

[ref18] Esser L.; Truong N. P.; Karagoz B.; Moffat B. A.; Boyer C.; Quinn J. F.; Whittaker M. R.; Davis T. P. Gadolinium-Functionalized Nanoparticles for Application as Magnetic Resonance Imaging Contrast Agents Via Polymerization-Induced Self-Assembly. Polym. Chem. 2016, 7 (47), 7325–7337. 10.1039/C6PY01797E.

[ref19] Liu N.; Marin R.; Mazouzi Y.; Cron G. O.; Shuhendler A.; Hemmer E. Cubic Versus Hexagonal—Effect of Host Crystallinity on the T 1 Shortening Behaviour of Nagdf 4 Nanoparticles. Nanoscale 2019, 11 (14), 6794–6801. 10.1039/C9NR00241C. 30907912

[ref20] Wang Y.-X. J.; Hussain S. M.; Krestin G. P. Superparamagnetic Iron Oxide Contrast Agents: Physicochemical Characteristics and Applications in Mr Imaging. Eur. Radiol. 2001, 11 (11), 2319–2331. 10.1007/s003300100908. 11702180

[ref21] Li J.; Li X.; Gong S.; Zhang C.; Qian C.; Qiao H.; Sun M. Dual-Mode Avocado-Like All-Iron Nanoplatform for Enhanced T1/T2Mri-Guided Cancer Theranostic Therapy. Nano Lett. 2020, 20 (7), 4842–4849. 10.1021/acs.nanolett.0c00817. 32578994

[ref22] Zhuang D.; Zhang H.; Hu G.; Guo B. Recent Development of Contrast Agents for Magnetic Resonance and Multimodal Imaging of Glioblastoma. J. Nanobiotechnol. 2022, 20 (1), 1–21. 10.1186/s12951-022-01479-6. PMC9204881 35710493

[ref23] Wang C.; Fan W.; Zhang Z.; Wen Y.; Xiong L.; Chen X. Advanced Nanotechnology Leading the Way to Multimodal Imaging-Guided Precision Surgical Therapy. Adv. Mater. 2019, 31 (49), 1904329 10.1002/adma.201904329. 31538379

[ref24] Shin T.-H.; Choi J.-s.; Yun S.; Kim I.-S.; Song H.-T.; Kim Y.; Park K. I.; Cheon J. T 1 and T 2 Dual-Mode Mri Contrast Agent for Enhancing Accuracy by Engineered Nanomaterials. ACS Nano 2014, 8 (4), 3393–3401. 10.1021/nn405977t. 24673493

[ref25] Choi J.-s.; Lee J.-H.; Shin T.-H.; Song H.-T.; Kim E. Y.; Cheon J. Self-Confirming “and” Logic Nanoparticles for Fault-Free MRI. J. Am. Chem. Soc. 2010, 132 (32), 11015–11017. 10.1021/ja104503g. 20698661 PMC2935492

[ref26] Duan B.; Wang D.; Wu H.; Xu P.; Jiang P.; Xia G.; Liu Z.; Wang H.; Guo Z.; Chen Q. Core–Shell Structurized Fe3o4@C@Mno2 Nanoparticles as Ph Responsive T1-T2* Dual-Modal Contrast Agents for Tumor Diagnosis. ACS Biomater. Sci. Eng. 2018, 4 (8), 3047–3054. 10.1021/acsbiomaterials.8b00287. 33435024

[ref27] Bai C.; Hu P.; Liu N.; Feng G.; Liu D.; Chen Y.; Ma M.; Gu N.; Zhang Y. Synthesis of Ultrasmall Fe3o4 Nanoparticles as T1–T2 Dual-Modal Magnetic Resonance Imaging Contrast Agents in Rabbit Hepatic Tumors. ACS Appl. Nano Mater. 2020, 3 (4), 3585–3595. 10.1021/acsanm.0c00306.

[ref28] Chen C.; Ge J.; Gao Y.; Chen L.; Cui J.; Zeng J.; Gao M. Ultrasmall Superparamagnetic Iron Oxide Nanoparticles: A Next Generation Contrast Agent for Magnetic Resonance Imaging. Wiley Interdiscip. Rev.: Nanomed. Nanobiotechnol. 2022, 14 (1), e1740 10.1002/wnan.1740. 34296533

[ref29] Rosensweig R. E. Heating Magnetic Fluid with Alternating Magnetic Field. J. Magn. Magn. Mater. 2002, 252, 370–374. 10.1016/S0304-8853(02)00706-0.

[ref30] Jiang G.; Fan D.; Tian J.; Xiang Z.; Fang Q. Self-Confirming Magnetosomes for Tumor-Targeted T1/T2 Dual-Mode Mri and Mri-Guided Photothermal Therapy. Adv. Healthcare Mater. 2022, 11 (14), 2200841 10.1002/adhm.202200841. 35579102

[ref31] Chou S.-W.; Chen T.-H.; Fa Y.-C.; Yang Y.-Y.; Lin C.-Y.; Kuo K.-L.; Wu S.-T.; Lin E. C.; Annie Ho J.-A.; Hsiao J.-K.; Chou P. T. Gadolinium-Engineered Magnetic Alloy Nanoparticles for Magnetic Resonance T1/T2 Dual-Modal and Computed Tomography Imaging. Chem. Mater. 2022, 34 (22), 10050–10058. 10.1021/acs.chemmater.2c02537.

[ref32] Thorat N. D.; Bohara R. A.; Yadav H. M.; Tofail S. A. Multi-Modal Mr Imaging and Magnetic Hyperthermia Study of Gd Doped Fe 3 O 4 Nanoparticles for Integrative Cancer Therapy. RSC Adv. 2016, 6 (97), 94967–94975. 10.1039/C6RA20135K.

[ref33] Santra S.; Jativa S. D.; Kaittanis C.; Normand G.; Grimm J.; Perez J. M. Gadolinium-Encapsulating Iron Oxide Nanoprobe as Activatable Nmr/Mri Contrast Agent. ACS Nano 2012, 6 (8), 7281–7294. 10.1021/nn302393e. 22809405 PMC3429787

[ref34] Liang C.; Li N.; Cai Z.; Liang R.; Zheng X.; Deng L.; Feng L.; Guo R.; Wei B. Co-Encapsulation of Magnetic Fe3O4 Nanoparticles and Doxorubicin into Biocompatible Plga-Peg Nanocarriers for Early Detection and Treatment of Tumours. Artif. Cells, Nanomed., Biotechnol. 2019, 47 (1), 4211–4221. 10.1080/21691401.2019.1687500. 31713444

[ref35] Stepanov A.; Fedorenko S.; Amirov R.; Nizameev I.; Kholin K.; Voloshina A.; Sapunova A.; Mendes R.; Rümmeli M.; Gemming T.; et al. Silica-Coated Iron-Oxide Nanoparticles Doped with Gd (Iii) Complexes as Potential Double Contrast Agents for Magnetic Resonance Imaging at Different Field Strengths. J. Chem. Sci. 2018, 130 (9), 1–10. 10.1007/s12039-018-1527-z.

[ref36] Yang M.; Gao L.; Liu K.; Luo C.; Wang Y.; Yu L.; Peng H.; Zhang W. Characterization of Fe3o4/Sio2/Gd2o (Co3) 2 Core/Shell/Shell Nanoparticles as T1 and T2 Dual Mode Mri Contrast Agent. Talanta 2015, 131, 661–665. 10.1016/j.talanta.2014.08.042. 25281156

[ref37] Bower D. V.; Richter J. K.; von Tengg-Kobligk H.; Heverhagen J. T.; Runge V. M. Gadolinium-Based MRI Contrast Agents Induce Mitochondrial Toxicity and Cell Death in Human Neurons, and Toxicity Increases with Reduced Kinetic Stability of the Agent. Invest. Radiol. 2019, 54 (8), 453–463. 10.1097/RLI.0000000000000567. 31265439

[ref38] Guardia P.; Di Corato R.; Lartigue L.; Wilhelm C.; Espinosa A.; Garcia-Hernandez M.; Gazeau F.; Manna L.; Pellegrino T. Water-Soluble Iron Oxide Nanocubes with High Values of Specific Absorption Rate for Cancer Cell Hyperthermia Treatment. ACS Nano 2012, 6 (4), 3080–3091. 10.1021/nn2048137. 22494015

[ref39] Lee S. H.; Kim B. H.; Na H. B.; Hyeon T. Paramagnetic Inorganic Nanoparticles as T1Mri Contrast Agents. Wiley Interdiscip. Rev.: Nanomed. Nanobiotechnol. 2014, 6 (2), 196–209. 10.1002/wnan.1243. 24123961

[ref40] Quarta A.; Rodio M.; Cassani M.; Gigli G.; Pellegrino T.; Del Mercato L. L. Multilayered Magnetic Nanobeads for the Delivery of Peptides Molecules Triggered by Intracellular Proteases. ACS Appl. Mater. Interfaces 2017, 9 (40), 35095–35104. 10.1021/acsami.7b05709. 28858466 PMC6091500

[ref41] Cabrera D.; Lak A.; Yoshida T.; Materia M.; Ortega D.; Ludwig F.; Guardia P.; Sathya A.; Pellegrino T.; Teran F. Unraveling Viscosity Effects on the Hysteresis Losses of Magnetic Nanocubes. Nanoscale 2017, 9 (16), 5094–5101. 10.1039/C7NR00810D. 28397910

[ref42] Zhang W.; Liu L.; Chen H.; Hu K.; Delahunty I.; Gao S.; Xie J. Surface Impact on Nanoparticle-Based Magnetic Resonance Imaging Contrast Agents. Theranostics 2018, 8 (9), 2521 10.7150/thno.23789. 29721097 PMC5928907

[ref43] Ni D.; Bu W.; Ehlerding E. B.; Cai W.; Shi J. Engineering of Inorganic Nanoparticles as Magnetic Resonance Imaging Contrast Agents. Chem. Soc. Rev. 2017, 46 (23), 7438–7468. 10.1039/C7CS00316A. 29071327 PMC5705441

[ref44] Mai B. T.; Conteh J. S.; Gavilán H.; Di Girolamo A.; Pellegrino T. Clickable Polymer Ligand-Functionalized Iron Oxide Nanocubes: A Promising Nanoplatform for ‘Local Hot Spots’ Magnetically Triggered Drug Release. ACS Appl. Mater. Interfaces 2022, 14 (43), 48476–48488. 10.1021/acsami.2c14752. 36256634 PMC9634696

[ref45] Gavilán H.; Simeonidis K.; Myrovali E.; Mazarío E.; Chubykalo-Fesenko O.; Chantrell R.; Balcells L.; Angelakeris M.; Morales M.; Serantes D. How Size, Shape and Assembly of Magnetic Nanoparticles Give Rise to Different Hyperthermia Scenarios. Nanoscale 2021, 13 (37), 15631–15646. 10.1039/D1NR03484G. 34596185

[ref46] Gutiérrez L.; De la Cueva L.; Moros M.; Mazarío E.; De Bernardo S.; De la Fuente J. M.; Morales M. P.; Salas G. Aggregation Effects on the Magnetic Properties of Iron Oxide Colloids. Nanotechnology 2019, 30 (11), 112001 10.1088/1361-6528/aafbff. 30609414

[ref47] Ovejero J. G.; Cabrera D.; Carrey J.; Valdivielso T.; Salas G.; Teran F. J. Effects of Inter-and Intra-Aggregate Magnetic Dipolar Interactions on the Magnetic Heating Efficiency of Iron Oxide Nanoparticles. Phys. Chem. Chem. Phys. 2016, 18 (16), 10954–10963. 10.1039/C6CP00468G. 27041536

[ref48] Estelrich i Latràs J.; Sánchez Martín M.; Busquets i Viñas M. Nanoparticles in Magnetic Resonance Imaging: From Simple to Dual Contrast Agents. Int. J. Nanomed. 2015, 10 (1), 1727–1741. 10.2147/IJN.S76501. PMC4358688 25834422

[ref49] Tromsdorf U. I.; Bigall N. C.; Kaul M. G.; Bruns O. T.; Nikolic M. S.; Mollwitz B.; Sperling R. A.; Reimer R.; Hohenberg H.; Parak W. J.; et al. Size and Surface Effects on the Mri Relaxivity of Manganese Ferrite Nanoparticle Contrast Agents. Nano Lett. 2007, 7 (8), 2422–2427. 10.1021/nl071099b. 17658761

[ref50] Zou Q.; Tang R.; Zhao H.-x.; Jiang J.; Li J.; Fu Y.-y. Hyaluronic-Acid-Assisted Facile Synthesis of Mnwo4 Single-Nanoparticle for Efficient Trimodal Imaging and Liver–Renal Structure Display. ACS Appl. Nano Mater. 2018, 1 (1), 101–110. 10.1021/acsanm.7b00047.

[ref51] Veiga M.; Mattiazzi P.; de Gois J. S.; Nascimento P. C.; Borges D. L.; Bohrer D. Presence of Other Rare Earth Metals in Gadolinium-Based Contrast Agents. Talanta 2020, 216, 120940 10.1016/j.talanta.2020.120940. 32456901

[ref52] Ling D.; Park W.; Park S.-j.; Lu Y.; Kim K. S.; Hackett M. J.; Kim B. H.; Yim H.; Jeon Y. S.; Na K.; Hyeon T. Multifunctional Tumor Ph-Sensitive Self-Assembled Nanoparticles for Bimodal Imaging and Treatment of Resistant Heterogeneous Tumors. J. Am. Chem. Soc. 2014, 136 (15), 5647–5655. 10.1021/ja4108287. 24689550

[ref53] Sivasubramanian M.; Chu C.-H.; Cheng S.-H.; Chen N.-T.; Chen C.-T.; Chuang Y. C.; Yu H.; Chen Y.-L.; Liao L.-D.; Lo L.-W. Multimodal Magnetic Resonance and Photoacoustic Imaging of Tumor-Specific Enzyme-Responsive Hybrid Nanoparticles for Oxygen Modulation. Front. Bioeng. Biotechnol. 2022, 10, 910902 10.3389/fbioe.2022.910902. 35910012 PMC9326367

[ref54] Lorkowski M. E.; Atukorale P. U.; Ghaghada K. B.; Karathanasis E. Stimuli-Responsive Iron Oxide Nanotheranostics: A Versatile and Powerful Approach for Cancer Therapy. Adv. Healthcare Mater. 2021, 10 (5), 2001044 10.1002/adhm.202001044. PMC7933107 33225633

[ref55] Low L. E.; Wu J.; Lee J.; Tey B. T.; Goh B.-H.; Gao J.; Li F.; Ling D. Tumor-Responsive Dynamic Nanoassemblies for Targeted Imaging, Therapy and Microenvironment Manipulation. J. Controlled Release 2020, 324, 69–103. 10.1016/j.jconrel.2020.05.014. 32423874

[ref56] Kang T.; Li F.; Baik S.; Shao W.; Ling D.; Hyeon T. Surface Design of Magnetic Nanoparticles for Stimuli-Responsive Cancer Imaging and Therapy. Biomaterials 2017, 136, 98–114. 10.1016/j.biomaterials.2017.05.013. 28525855

[ref57] Ellis C. M.; Pellico J.; Davis J. J. Magnetic Nanoparticles Supporting Bio-Responsive T 1/T 2 Magnetic Resonance Imaging. Materials 2019, 12 (24), 4096 10.3390/ma12244096. 31817929 PMC6947368

[ref58] Avugadda S. K.; Wickramasinghe S.; Niculaes D.; Ju M.; Lak A.; Silvestri N.; Nitti S.; Roy I.; Samia A. C. S.; Pellegrino T. Uncovering the Magnetic Particle Imaging and Magnetic Resonance Imaging Features of Iron Oxide Nanocube Clusters. Nanomaterials 2021, 11 (1), 62 10.3390/nano11010062. PMC7824301 33383768

[ref59] Hingorani D. V.; Bernstein A. S.; Pagel M. D. A Review of Responsive Mri Contrast Agents: 2005–2014. Contrast Media Mol. Imaging 2015, 10 (4), 245–265. 10.1002/cmmi.1629. 25355685 PMC4414668

[ref60] Dai Y.; Su J.; Wu K.; Ma W.; Wang B.; Li M.; Sun P.; Shen Q.; Wang Q.; Fan Q. Multifunctional Thermosensitive Liposomes Based on Natural Phase-Change Material: Near-Infrared Light-Triggered Drug Release and Multimodal Imaging-Guided Cancer Combination Therapy. ACS Appl. Mater. Interfaces 2019, 11 (11), 10540–10553. 10.1021/acsami.8b22748. 30807086

[ref61] Guardia P.; Riedinger A.; Nitti S.; Pugliese G.; Marras S.; Genovese A.; Materia M. E.; Lefevre C.; Manna L.; Pellegrino T. One Pot Synthesis of Monodisperse Water Soluble Iron Oxide Nanocrystals with High Values of the Specific Absorption Rate. J. Mater. Chem. B 2014, 2 (28), 4426–4434. 10.1039/c4tb00061g. 32261543

[ref62] Connord V.; Mehdaoui B.; Tan R. P.; Carrey J.; Respaud M. An Air-Cooled Litz Wire Coil for Measuring the High Frequency Hysteresis Loops of Magnetic Samples—a Useful Setup for Magnetic Hyperthermia Applications. Rev. Sci. Instrum. 2014, 85 (9), 093904 10.1063/1.4895656. 25273736

[ref63] Mehdaoui B.; Carrey J.; Stadler M.; Cornejo A.; Nayral C.; Delpech F.; Chaudret B.; Respaud M. Influence of a Transverse Static Magnetic Field on the Magnetic Hyperthermia Properties and High-Frequency Hysteresis Loops of Ferromagnetic Feco Nanoparticles. Appl. Phys. Lett. 2012, 100 (5), 052403 10.1063/1.3681361.

[ref64] Cabrera D.; Coene A.; Leliaert J.; Artes-Ibanez E. J.; Dupré L.; Telling N. D.; Teran F. J. Dynamical Magnetic Response of Iron Oxide Nanoparticles inside Live Cells. ACS Nano 2018, 12 (3), 2741–2752. 10.1021/acsnano.7b08995. 29508990

