# Supplementary material for: Protease-Mediated T1 Contrast Enhancement of Multilayered Magneto-Gadolinium Nanostructures for Imaging and Magnetic Hyperthermia
Source: ACS Appl Mater Interfaces. 2024 Jan 31;16(6):6743–55. doi: 10.1021/acsami.3c13914 (PMC10875642; doi:10.1021/acsami.3c13914)
Supplement: Supplementary file 1 — am3c13914_si_001.pdf [file am3c13914_si_001.pdf]

# Supporting Information

# Protease-mediated $T_1$ Contrast Enhancement of Multilayered Magneto-Gadolinium nanostructures for Imaging and Magnetic Hyperthermia

Sahitya Kumar Avugadda[1$], Nisarg Soni[1$], Emille M. Rodrigues[1], Stefano Persano[1] and Teresa Pellegrino[1]*

[a]Nanomaterials for Biomedical Applications, Istituto Italiano di Tecnologia, 16163 Genova, Italy

*Corresponding author: teresa.pellegrino@iit.it

$ these authors have contributed equally to this work

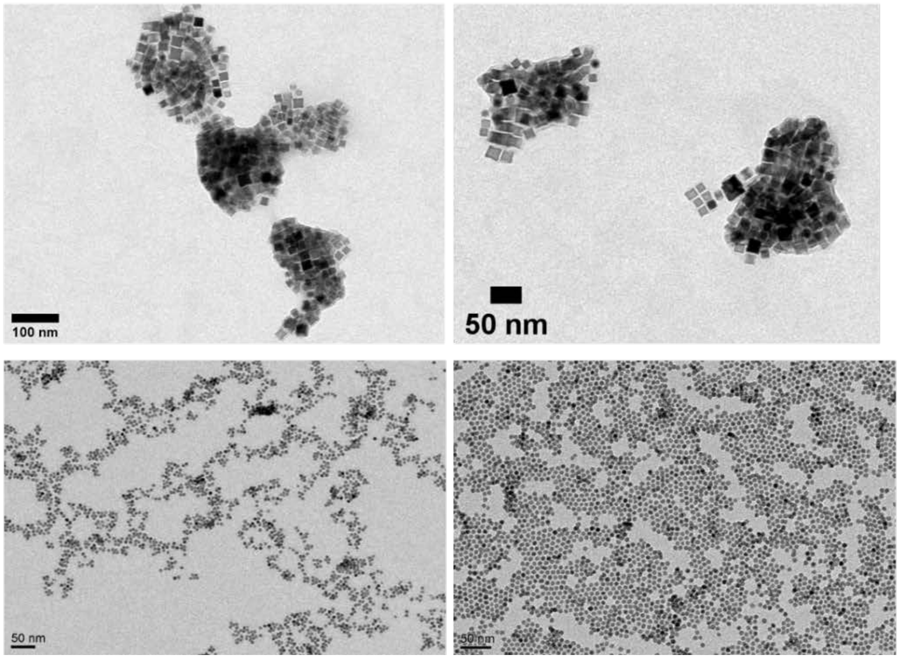


Figure S1. TEM images of magnetic nanobeads (top images) and polyacrylic acid-coated gadolinium nanoparticles (PAA-GdNPs (lower images, 50 nm scale bar) dropped from aquoeus solution after water solubilization.

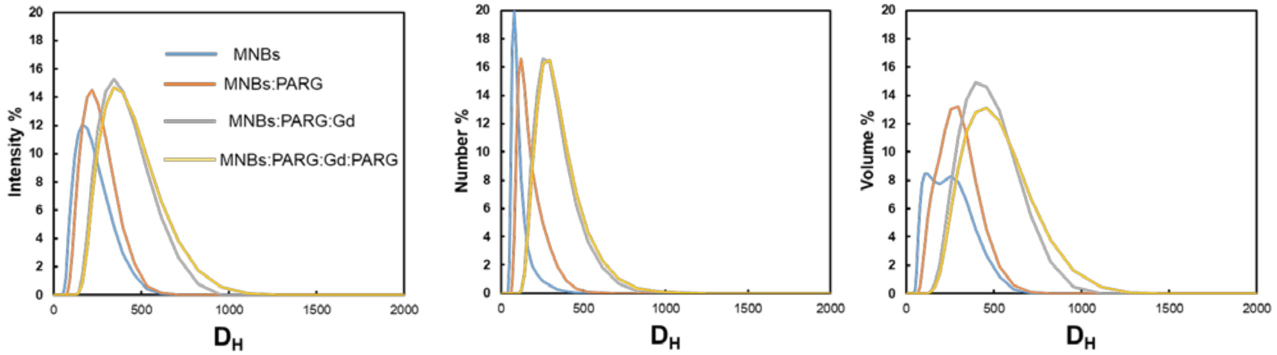


Figure S2. Dynamic light scattering peaks of MNBs, MNB:PARG, MNBs:PARG:Gd and MNBs:PARG:Gd:PARG nanostructures at each stage of layering in water, peaks are presented in terms of number, intensity and volume percent.

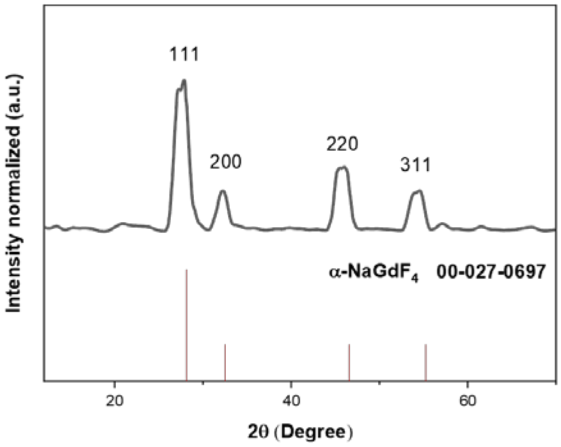


Figure S3: Powder-XRD pattern of the $NaGdF_4$ nanoparticles (NPs) showing that the sample is composed of pure-phase cubic $NaGdF_4$ matching the standard PDF 00-027-0697. Sample was prepared by drop-casting 20 microliters of the chloroform suspension of $NaGdF_4$ NPs on a silicon wafer and measurements were performed on the powder after the sample was dried.

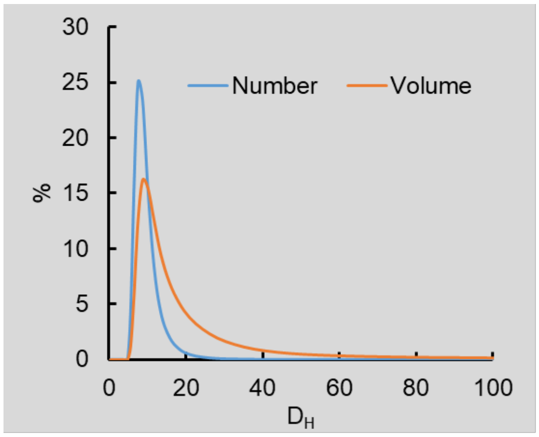


| PAA-Gd NPs | $D_H$ | PDI |
|---|---|---|
| Number | 8.9 ± 1 nm | 0.390 |
| Intensity | 216±96 nm | |
| Volume | 55±34 nm | |

Figure S4. Dynamic light scattering peaks; by volume and intensity for PAA-Gd NPs.

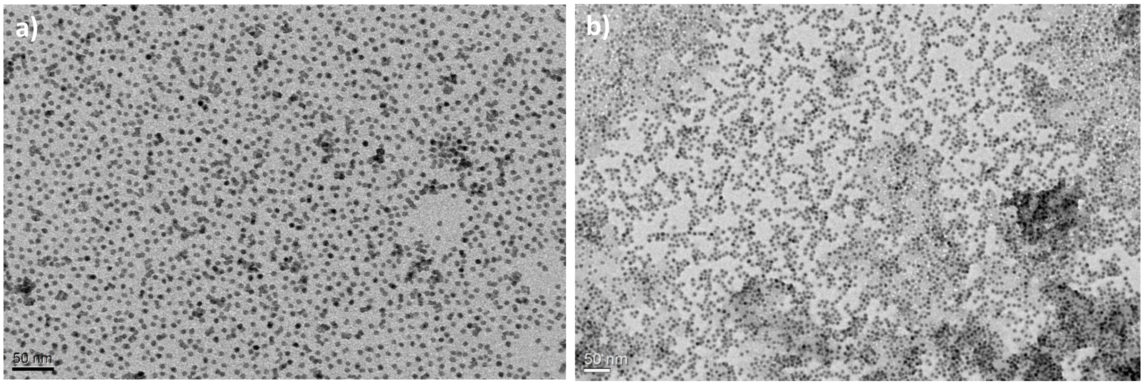


Figure S5. a) TEM images of water-soluble Gd NPs coated with a catechol-based PEG-coated polymer (CAPEG-Gd NPs), dropped and dried on a carbon-coated copper grid. b) TEM image of PAA-Gd NPs coated with PARG (PAA-GdNPs:PARG).

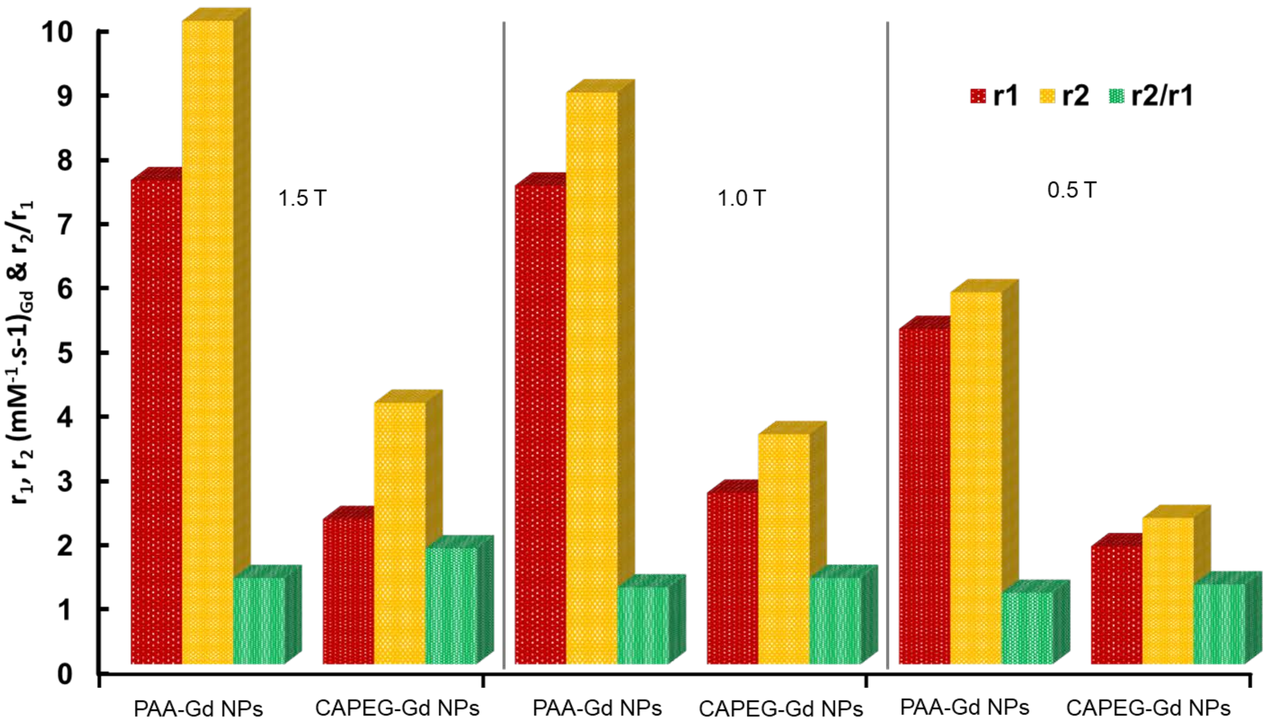


Figure S6. The histograms comparing the relaxation rates of the Gd NPs coated with Cathacol-based PEG polymer (CAPEG-Gd NPs) and PAA-coated Gd NPs.

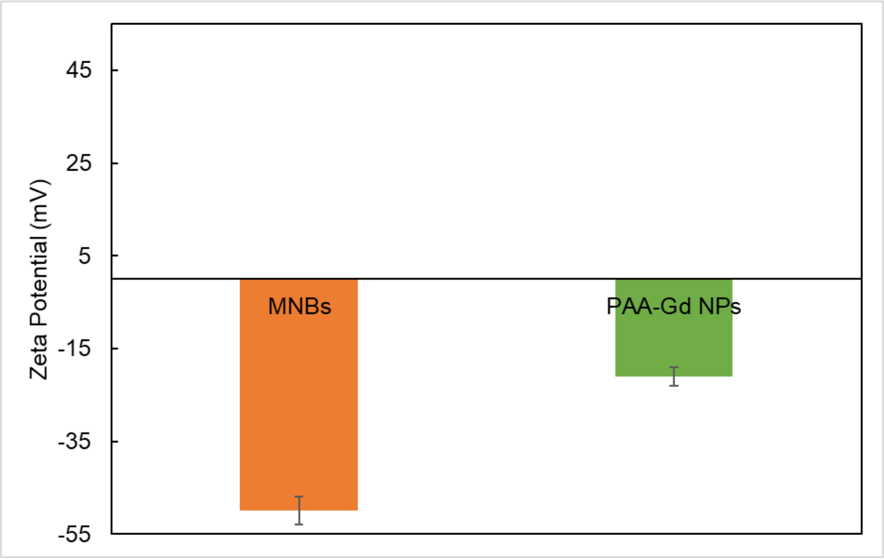


Figure S7. Surface zeta potential of magnetic nanobeads and polyacrylic acid-coated PPA-Gd NPs in water.

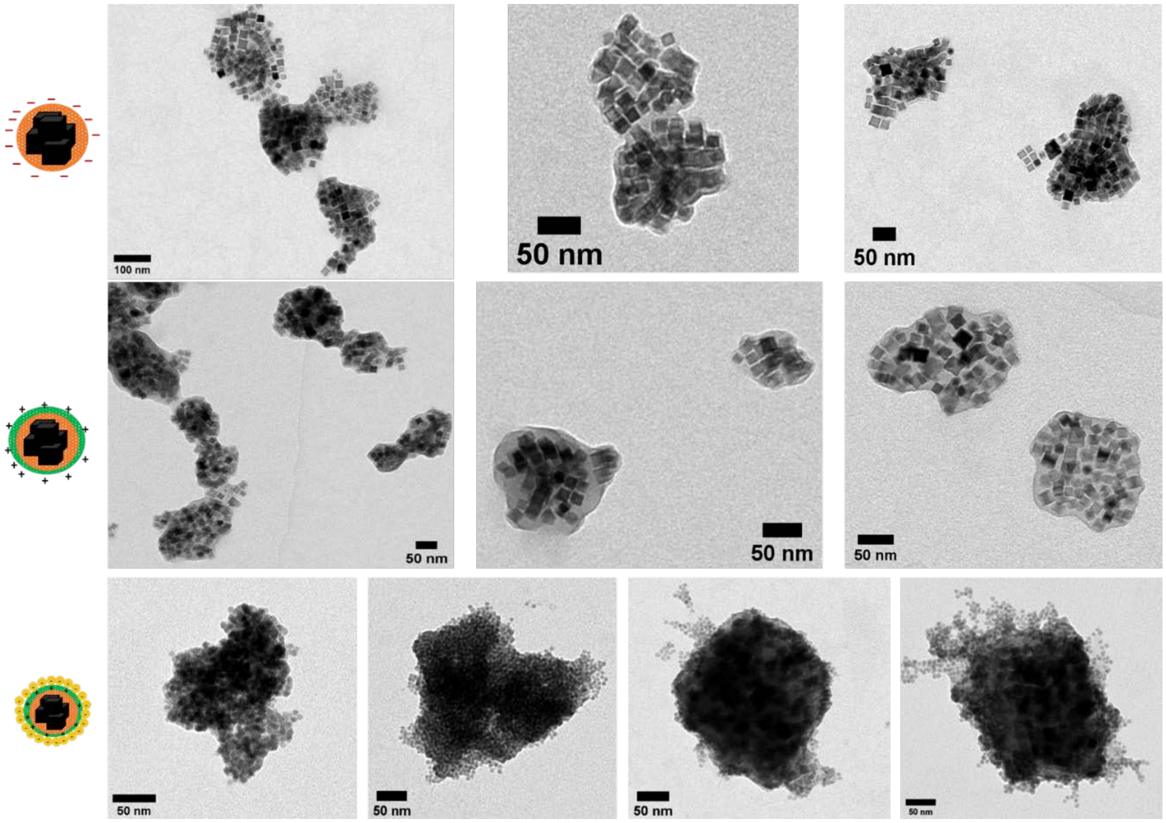


Figure S8. TEM images of water dropped a) pure MNBs before layering, b) the same beads after first layering of PARG (MNB:PARG), and c) the same beads of MNB:PARG loading with PAA-Gd NPs (MNB:PARG:Gd).

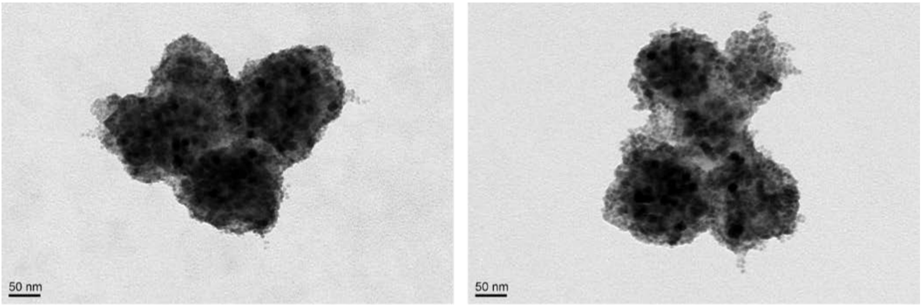


Figure S9. TEM image of MNBs after completion of layering process with Gd NPs (MNB:PARG:Gd:PARG).

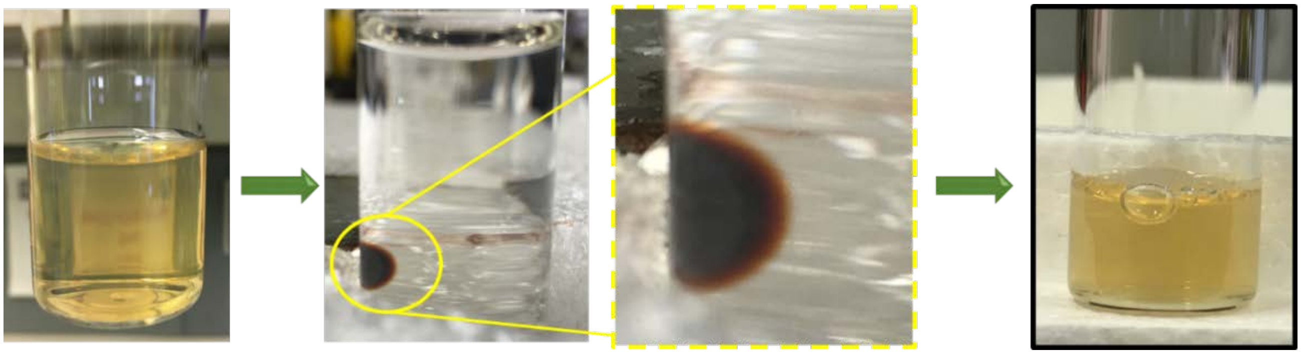

Figure S10. Photographs demonstrating the reversible magnetic accumulation properties of MNB:PARG:Gd:PARG.

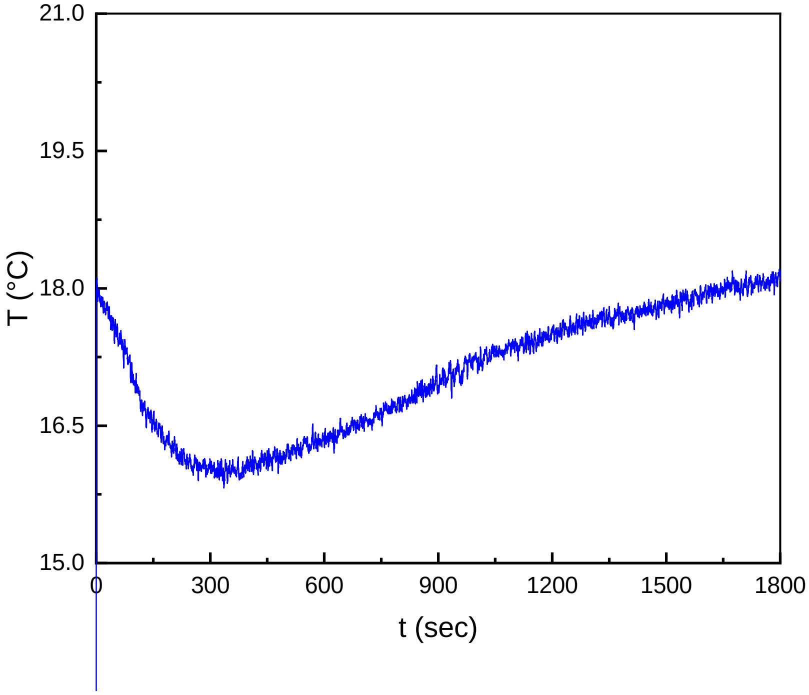


Figure S11: temperature versus time curve of a water volume of 180 µL exposed to AMF of 180 kHz frequency and 30 kA/m field intensity. Under these field parameters, no change in temperature is observed for plain water within 30 minutes exposure.

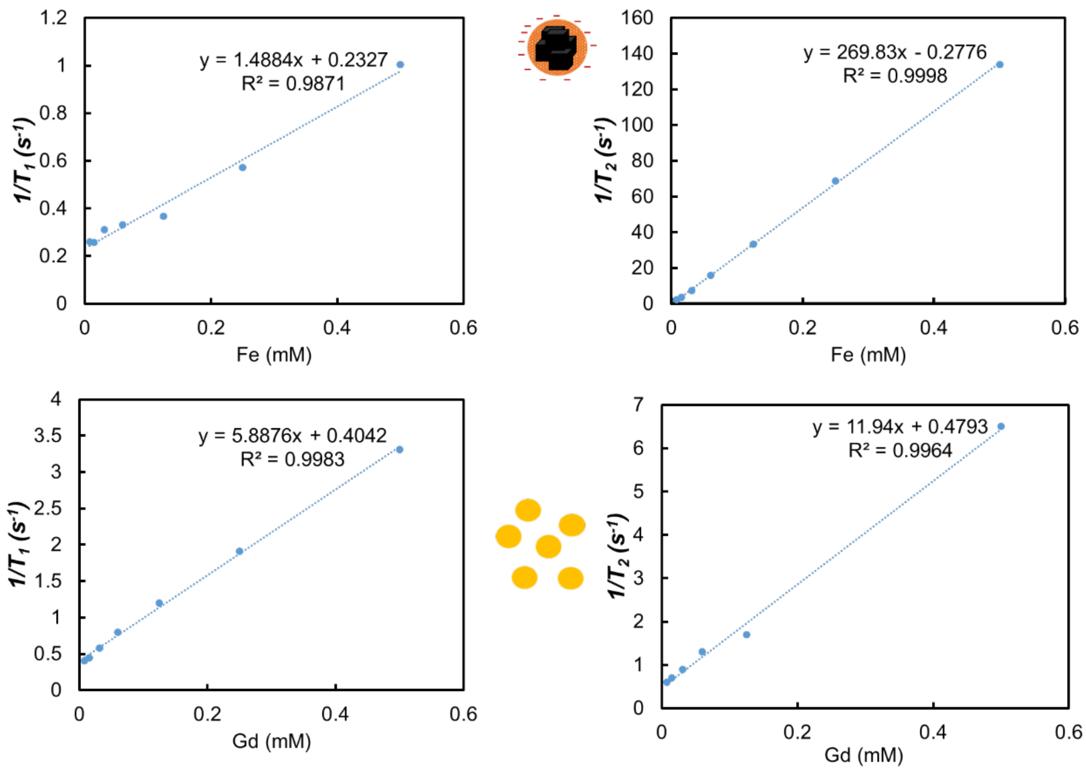


Figure S12. Linear calibration curves of MNBs (upper row) and PAA-Gd NPs (lower row) in 0.5 % agarose solution, whose inverse of relaxation times ($1/T_1$ or $1/T_2$) were plotted against the concentration of Fe and Gd respectively.

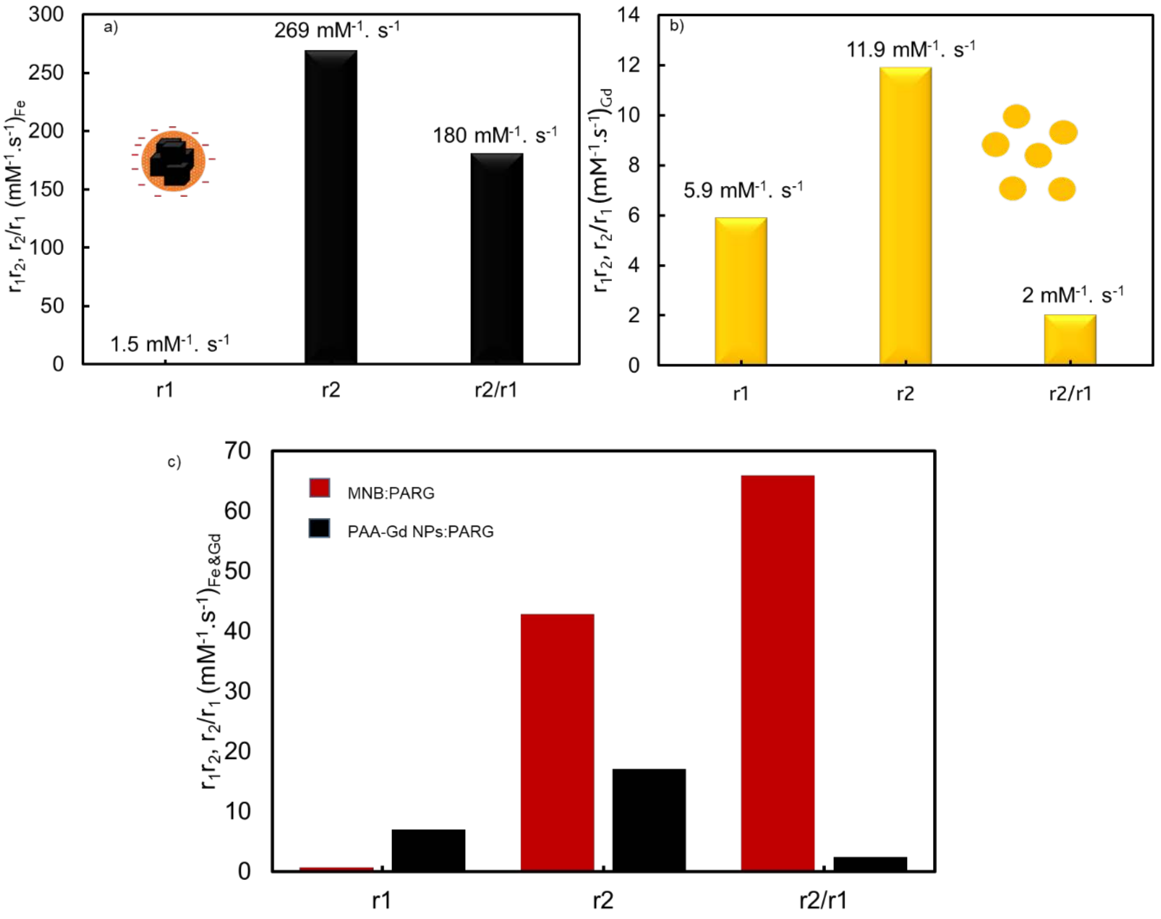


Figure S13. The relavities; $r_1$, $r_2$, and $r_2/r_1$ ratios, of a,) Magnetic nanobeads and b) PAA-Gd NPs in the purest form, independently measured before assembling. C) comparison of relaxivities of MNBs (MNB:PARG) and PAA-Gd NPs (PAA-PARG) after coating just with PARG.

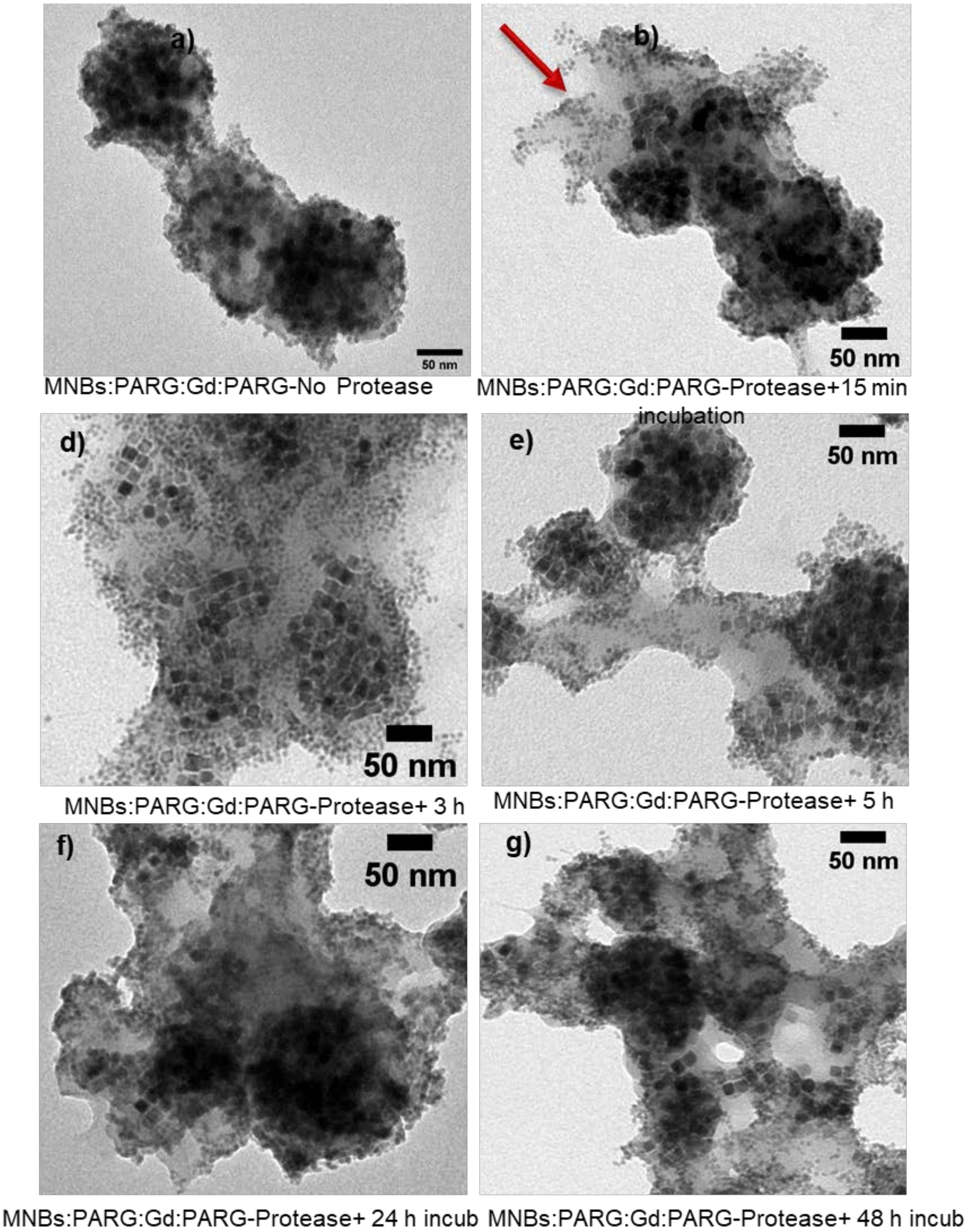


Figure S14. Micrographs of the magneto-gadolinium nanostructures, before and after exposure to protease enzyme, at various time points of incubation starting from no enzyme to 0.25, 3, 5, 24, and 48 hours of incubation with protease at 37 °C.

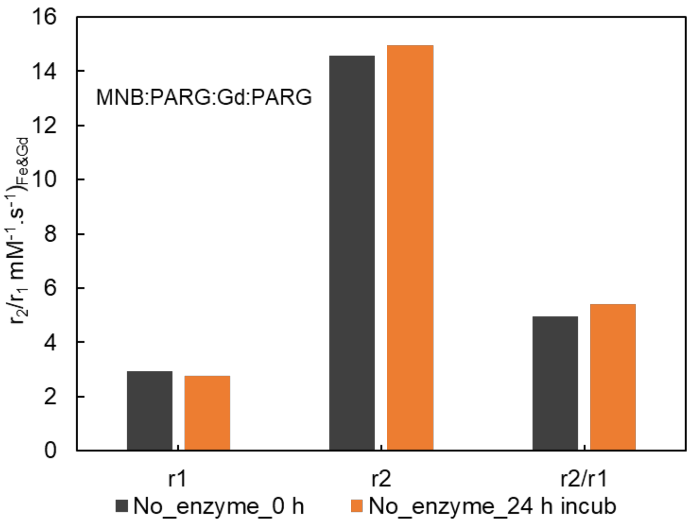


Figure S15. The comparison of $r_1$, $r_2$ and $r_2/r_1$ rate values of magneto-gadolinium nanostructures (MNB:PARG:Gd:PARG) before and after incubating at 37 °C without any addition of enzyme. This comparison emphasizes that the 37 °C incubation has almost no impact on the degradation of the polymer, hence, on the relaxivity changes.

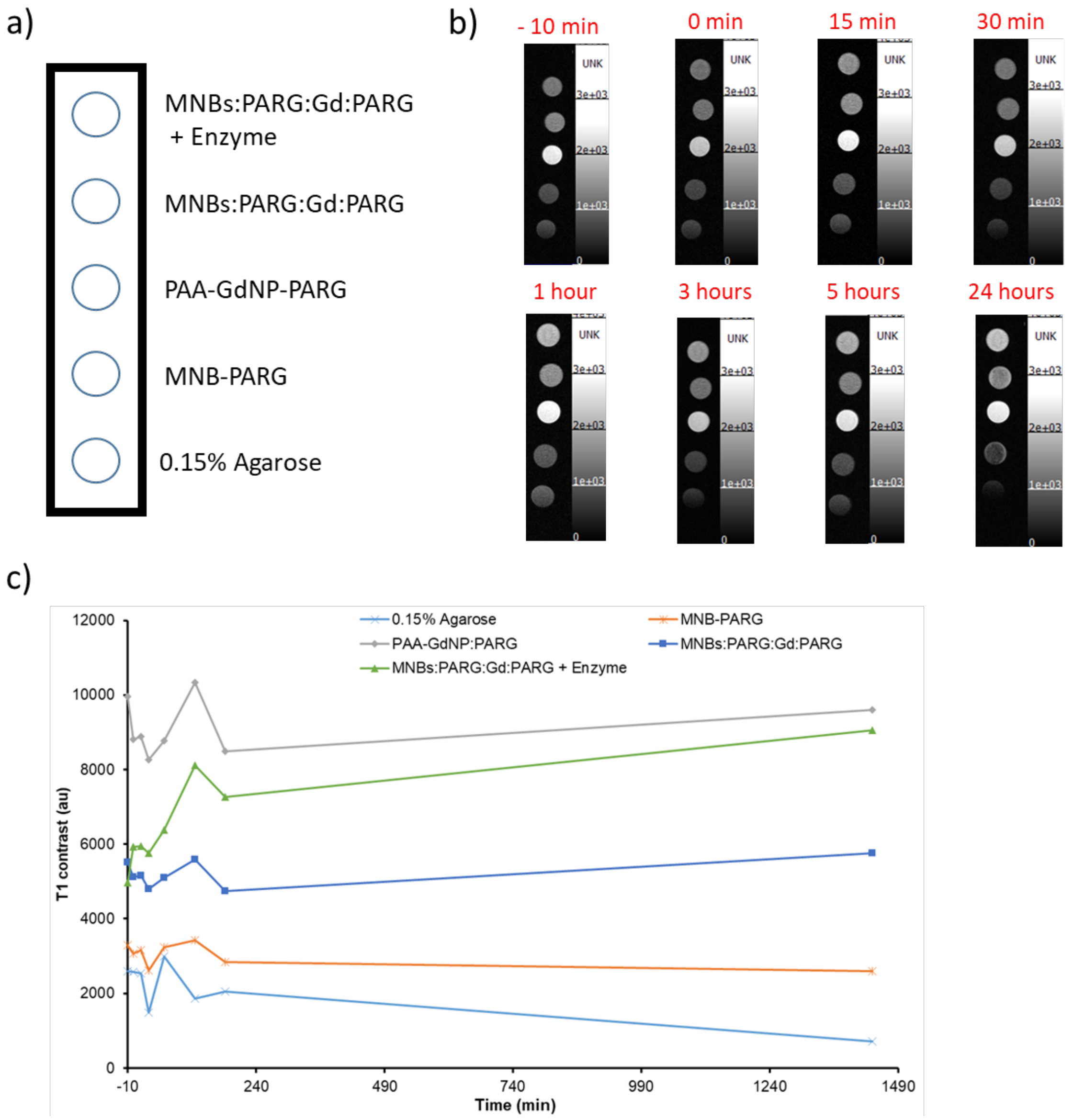


Figure S16. a) The sequence of the tubes containing different NP solutions to be used for MR imaging. b) 3T MR images were acquired at different time points as mentioned. c) T1 contrast plots of signals as obtained from the 3T MR acquisitions.

| Area (mJ/kg) | | | | |
|---|---|---|---|---|
| 240 kHz | | | | |
| kA/m | MNBs | Std.Dev | MNB:PARG:Gd:PARG | Std.Dev |
| 24 | 1132.1 | 49.5547 | 961.072 | 59.409 |
| 20 | 680.221 | 62.3946 | 550.551 | 14.996 |
| 16 | 386.175 | 12.0678 | 311.364 | 18.519 |
| 12 | 158.323 | 5.86863 | 124.323 | 36.045 |
| 150 kHz | | | | |
| 24 | 1129.03 | 59.3457 | 849.267 | 14.85 |
| 20 | 721.834 | 59.4326 | 589.677 | 6.9174 |
| 16 | 326.193 | 27.7165 | 308.694 | 24.581 |
| 12 | 160.325 | 13.1524 | 91.3242 | 15.711 |

Table S1. Absolute area values of AC hysteresis loops corresponding to the Magnetic nanobeads and MNB:PARG:Gd:PARG, under 240 kHz and 150 kHz, within a field range of 24 kA/m to 12 kA/m.

| M_max (Am^2/kg | | | | |
|---|---|---|---|---|
| kA/m | MNBs | Std.Dev | MNB:PARG:Gd:PARG | Std.Dev |
| 240 kHz | | | | |
| 24 | 70.3217 | 2.44461 | 69.2301 | 1.59627 |
| 20 | 62.0488 | 1.99713 | 63.1751 | 3.8262 |
| 16 | 33.7711 | 7.29397 | 45.1667 | 1.77 |
| 12 | 28.3017 | 1.90435 | 26.3909 | 3.84418 |
| 150 kHz | | | | |
| 24 | 73.2877 | 4.93682 | 70.3954 | 2.95185 |
| 20 | 53.5658 | 9.50549 | 55.2523 | 1.41158 |
| 16 | 37.839 | 3.29635 | 42.382 | 4.41378 |
| 12 | 28.2216 | 2.17612 | 30.8508 | 2.1226 |

Table S2. Saturation magnetization values obtained from Ac hysteresis loops corresponding to the Magnetic nanobeads and MNB:PARG:Gd:PARG, under 240 kHz and 150 kHz, within a field range of 24 kA/m to 12 kA/m.

| | $R^2$- Fit values | |
|---|---|---|
| | $1/T_1$ | $1/T_2$ |
| No Enzyme | 0.9974 | 0.9798 |
| 3 min | 0.978 | 0.968 |
| 1 hour | 0.9907 | 0.9594 |
| 3 hours | 0.995 | 0.9875 |
| 5 hours | 0.9925 | 0.9954 |
| 24 hours | 0.989 | 0.9967 |

Table S3. Values in the table are the correlation coefficient, $R^2$-fit values obtained from the linear plots of $1/T_1$ (in Figure 5d) and $1/T_2$ versus concentrations (Figure 5e) for the sample MNBs:PARG:Gd:PARG.
